# Engineering mesenchymal stem cells for premature ovarian failure: overcoming challenges and innovating therapeutic strategies

**DOI:** 10.7150/thno.102641

**Published:** 2024-10-07

**Authors:** Zijun Yuan, Yinping Zhang, Xinyu He, Xiang Wang, Xingyue Wang, Siqi Ren, Jiahong Su, Jing Shen, Xiang Li, Zhangang Xiao

**Affiliations:** 1 Laboratory of Molecular Pharmacology, Department of Pharmacology, School of Pharmacy, Southwest Medical University, Luzhou, China.; 2 Sichuan College of Traditional Chinese Medicine, Sichuan Mianyang 621000, China.; 3 Cell Therapy & Cell Drugs of Luzhou Key Laboratory, Luzhou, Sichuan, China.; 4 Department of Pharmacology, School of Pharmacy, Sichuan College of Traditional Chinese Medicine, Sichuan Mianyang 621000, China.; 5 Luzhou People's Hospital, Luzhou, Sichuan, China.

**Keywords:** mesenchymal stem cells, premature ovarian failure, heterogeneity, genetic engineering, tissue engineering

## Abstract

Premature ovarian failure (POF) is a leading cause of infertility in women, causing significant psychological and physical distress. Current therapeutic options are limited, necessitating the exploration of new treatments. Mesenchymal stem cells (MSCs), known for their remarkable homing and regenerative properties, have emerged as a promising intervention for POF. However, their clinical efficacy has been inconsistent. This paper aims to address these challenges by examining the cellular heterogeneity within MSC populations, which is crucial for identifying and selecting specific functional subpopulations for clinical applications. Understanding this heterogeneity can enhance therapeutic efficacy and ensure treatment stability. Additionally, this review comprehensively examines the literature on the effectiveness, safety, and ethical considerations of MSCs for ovarian regeneration, with a focus on preclinical and clinical trials. We also discuss potential strategies involving genetically and tissue-engineered MSCs. By integrating insights from these studies, we propose new directions for the design of targeted MSC treatments for POF and related disorders, potentially improving outcomes, addressing safety concerns, and expanding therapeutic options while ensuring ethical compliance.

## 1. Introduction

Premature ovarian failure (POF) is an early menopause phenomenon occurring in women under 40 due to declining ovarian function 1. POF is a multifactorial disorder with high incidence. The main clinical manifestation includes scanty or absent menstruation for at least 4 months, with FSH (follicle-stimulating hormone) levels ≥25 IU/L in two random tests four weeks apart, accompanied by decreased estrogen levels 2. POF is one of the most common causes of female infertility 3. Its prevalence is 0.01% among women aged 20, 0.1% at 30 years, and 1% at 40 years 4. Although its etiology is complex and unclear, confirmed causes include genetic factors, autoimmune diseases, iatrogenic damage from chemotherapy, and enzymatic defects 5. Unfortunately, therapeutic options for diagnosed POF patients are limited. Hormone replacement therapy (HRT) is the mainstay but carries an increased risk of cancer 6. Overall, due to its diverse etiology and clinical manifestation, treating POF remains a significant challenge.

Mesenchymal stem cells (MSCs), as pluripotent cells with self-renewal and multi-lineage differentiation capacities, play a key role in tissue healing and regenerative medicine 7. MSCs are easily obtainable and exhibit low immunogenicity 8, harvested from various adult tissues such as bone marrow (BM), umbilical cord (UC), placenta, amniotic membrane, amniotic fluid, peripheral blood, adipose tissue, and menstrual fluid , making them excellent sources of growth factors or cytokines. Furthermore, MSCs possess homing abilities, meaning they can migrate to injury sites, differentiate into local components of the injured area, and secrete chemotactic factors, cytokines, and growth factors conducive to tissue regeneration 9, suggesting a broad potential application for MSCs in the field of POF 10.

Existing research demonstrates the significant therapeutic effect of MSCs in treating POF. For instance, a clinical application of autologous MSCs in patients with idiopathic POF showed that 2 cases (20% in totality) resumed menstruation three months post-transplantation, with one case (10% in totality) even achieving pregnancy and delivering a healthy baby 6. However, despite MSCs displaying potent therapeutic effects, their stability and uniformity of outcome remain less than ideal. While factors inherent to the condition of POF may play a role, a more significant issue is the heterogeneity of MSCs, which limits their further clinical application due to their mixture of functionally diverse subpopulations 11. Currently, there's no clear definition for these stem cell subpopulations, making it challenging to purify and isolate them to enhance treatment stability 12. In addition to addressing these challenges, this paper also focuses on the safety and ethical concerns surrounding the use of MSCs in POF therapy, as well as ongoing preclinical and clinical trials aimed at validating their therapeutic potential. Nonetheless, we find that genetic and tissue engineering modifications of MSCs can further improve their inherent characteristics, such as homing, differentiation, and cytokine secretion, as well as endow them with new functions, such as serving as carriers or therapeutic switches, thereby enhancing their therapeutic effect.

Therefore, identifying MSC subpopulations, clarifying their distinct functions, exploring their relationships, and engineering them, holds significant importance for both basic research and clinical application of MSCs. This paper explores potential ways to enhance the clinical efficacy of MSCs, primarily by revealing the common characteristics of different functional MSC subpopulations to provide new perspectives for the clinical selection of high-quality subpopulations, thereby helping to stabilize treatment effectiveness, and by reviewing advanced techniques in MSC engineering modifications to offer new insights into enhancing MSC therapeutic effects, while carefully considering safety, efficacy, and ethical implications.

## 2. Factors leading to the variability in MSCs therapy outcomes

**Figure 1:**
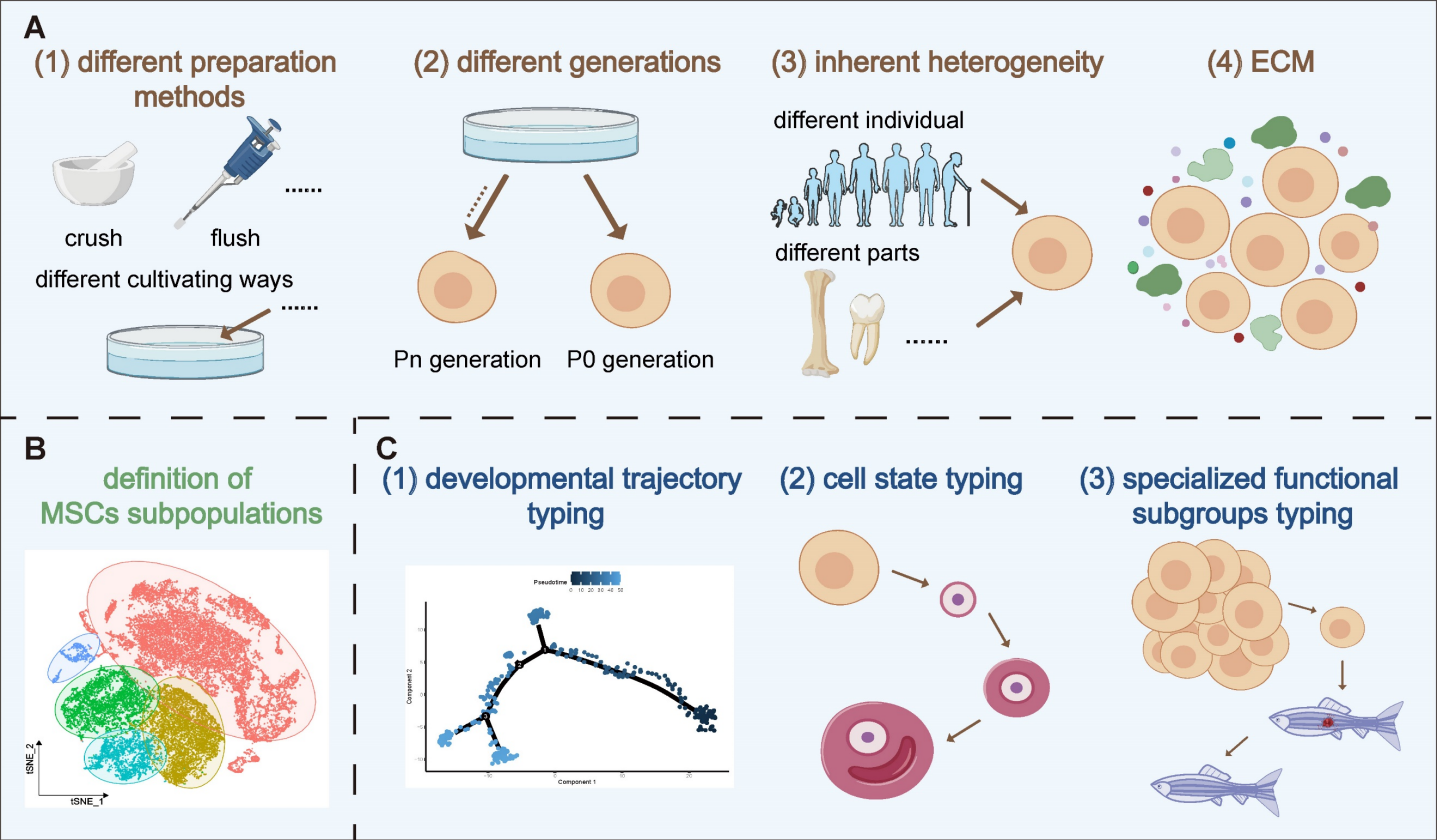
** Factors contributing to the erratic efficacy of MSCs.** (A) The instability in MSC efficacy is primarily due to batch effects caused by varying preparation methods and passages, intrinsic heterogeneity of MSCs, and their ECM. (B) The nomenclature heterogeneity of MSCs is examined. (C) Single-cell techniques are employed to analyze the molecular characteristics of MSCs, focusing on developmental trajectory typing, cell state typing, and cell-specific functional subpopulation typing.

Variability in MSC therapy outcomes is influenced by batch effects, inherent heterogeneity and ECM. The impact of batch effects on the consistency of MSC therapy stems from variations in differences in preparation and culture techniques or different passages. For example, Chiara *et al.* found that methods like flushing, crushing, and enzymatic digestion release distinct cell groups, such as fibroblasts and Schwann cells 13. MSCs can also develop subpopulations with different morphologies and functions during *in vitro* culture, even under controlled conditions 11. Factors like inflammatory stimuli can further modify MSC characteristics, enhancing their immunomodulatory effects 14. Additionally, the use of cryopreserved MSCs in clinical settings, as opposed to fresh or cultured MSCs, introduces variability, as freezing alters cytokine signaling, cell proliferation, and apoptosis levels. Medrano-Trochez *et al.* noted significant functional differences between MSCs before and after freezing 14. Passage number is another critical factor affecting therapeutic outcomes. Zhang *et al.* found substantial gene expression disparities between primary and cultured MSCs, with primary MSCs linked to ECM organization, collagen biosynthesis, and vascular development, while cultured MSCs activated the P53 pathway, indicating a "proliferation-differentiation-aging" process 15. Xie's scRNA-seq analysis showed that early passage MSCs (P1) had superior proliferation and adipogenic potential, whereas later passages (P3) excelled in osteogenic differentiation and immune regulation 16. Other studies highlighted that markers like CD146 and DNA methylation patterns differ post-culture, with primary BM-MSCs exhibiting stronger hematopoietic support and homing efficiency than their cultured counterparts 17. These findings emphasize that passage number is crucial in MSC cultivation, significantly impacting therapeutic (Figure 1A).

Beyond batch effects, inherent factors like the cell donor, age variations and source location, as well as heterogeneity induced by the microenvironment, significantly impact the stability of MSC efficacy 14, 18. For instance, studies show MSCs from different donors or tissue sources, such as umbilical cord, amniotic fluid, and bone marrow, possess varying differentiation capacities and functional characteristics 19. Even within the same tissue, MSCs derived from different locations, such as femur versus iliac crest, display distinct differentiation potentials 20, 21. Interestingly, MSC subpopulation heterogeneity might have a more significant impact on therapeutic outcomes than individual donor differences 22. Research indicates that markers like EGFR-3 and Ang-1 in BM-MSCs show little correlation with donor age or gender but are crucial for MSC function 23.

The ECM, a critical component of the cellular microenvironment, also plays a significant role in influencing MSC behavior and contributing to their heterogeneity 24. While cell cycle stages have been proposed as a source of MSC diversity before 25, 26, recent studies suggest that ECM-related factors, rather than cell cycle differences, are more critical in driving heterogeneity 26. The ECM influences MSC immunosuppressive functions and niche integrity, which can affect aging-related phenotypes. Despite these complexities, certain MSC types, such as those derived from umbilical cords, demonstrate relatively consistent behavior during *in vitro* expansion, highlighting their potential for standardized therapeutic applications 26.

In conclusion, the variability in MSC therapy outcomes is driven by a combination of preparation methods, passage numbers, and inherent heterogeneity influenced by factors like the ECM. The use of advanced technologies like scRNA-seq provides deeper insights into MSC heterogeneity, offering the potential for more targeted and consistent therapeutic applications (Figure 1A).

## 3. Exploring MSC heterogeneity using scRNA-seq technology

Although preliminary evidence exists regarding the clinical safety and efficacy of MSC therapy, its pronounced heterogeneity leaves the mechanisms of action and key characteristics largely unknown, leading to variable clinical outcomes and poor reproducibility. This constitutes a significant barrier to successful clinical translation 27. Studies have shown that the expression of many cell surface markers commonly used for MSC sorting changes before and after cultivation, suggesting that relying solely on *in vitro* culture and identification by cell surface antigens may result in marking heterogeneous groups rather than specific cell types 15. Furthermore, the lack of a precise definition for the composition types of MSCs impedes the ability to accurately predict and control the behavior of these heterogeneous cell populations, thus obstructing the large-scale standardization and procedural application of MSCs in clinical translation. Therefore, to gain a comprehensive understanding of MSC functional subpopulations, we first review the use of scRNA-seq to identify cell clusters with similar characteristics, thereby precisely defining MSC functional subpopulations. Subsequently, by identifying tissue-specific MSC subpopulations and gene expression characteristics, we aim to isolate cell subpopulations with specific functions, enhancing the resource value of MSC subpopulations and facilitating the standardization and therapeutic application of MSC products.

### 3.1. Defining MSC subpopulations using scRNA-seq technology

The advent of scRNA-seq has enabled the precise identification of corresponding cell subpopulations in humans and other species. Lineage tracing studies have consistently demonstrated common surface phenotypes of MSCs. However, comparative analyses of scRNA-seq datasets have revealed additional nomenclature heterogeneity, meaning that even within the same species, different research groups may assign different names to overlapping cell clusters based on their research focus. This leads to some degree of deviation in cell identity, creating a false impression of many cell clusters with different characteristics 28. For example, based on the expression of differentiation genes, Hou *et al.* analyzed single-cell data from MSCs derived from four representative tissue sources: UC, BM, synovial tissue, and adipose tissue, identifying three main subpopulations: osteogenic MSCs, chondrogenic MSCs, and adipogenic/myogenic MSCs 29. Based on ECM expression, Wang *et al.* identified seven tissue-specific subpopulations and five conserved subpopulations from MSCs from multiple tissue sources 30. Based on subpopulation identification and signaling pathway activation, Jia *et al.* identified specific subpopulations in MSCs derived from the human UC and human synovium, finding eleven subpopulations in UC-derived MSCs and seven in synovial-derived MSCs 31. Based on previously characterized BM stroma gene expression patterns, Wolock *et al.* identified specific subpopulations of non-hematopoietic cells in the BM, including multipotent stromal cells, adipocytes, and chondrocytes, even when all are based on the expression of subpopulation biological functions, different research groups have different definitions of functional subpopulations 32. Chen *et al.* dissected different molecular spectra of WJ-MSC populations cultured from different donors, identifying four functional subpopulations: Proliferative MSCs (high proliferative potential), niche-supporting_MSCs (rich in ECM-related molecules), metabolism-related_MSCs (related to metabolic capacity) and biofunctional-type_MSCs (promoting regeneration and immune regulation). Among them, proliferative MSCs were the most numerous group, playing a central role in cell growth and development; niche-supporting MSCs were central to MSCs, their integrity mainly influenced by cell-matrix dynamics and ECM remodeling; biological function-type MSCs highly expressed immune-related and angiogenesis-promoting genes 33. Xie *et al.* identified three MSC functional subpopulations: CD26 stemness subpopulation, CMKLR1 functional subpopulation, and proliferative subpopulation, with the CMKLR1 functional subpopulation displaying stronger immunomodulatory and osteogenic differentiation capabilities but lower adipogenic differentiation and proliferation potential 16.

In summary, the introduction of scRNA-seq technology has provided a new breakthrough for precisely identifying MSC subpopulations. However, the exposure of the problem of nomenclature heterogeneity also suggests that more unified and accurate standards still need to be reached in the identification and functional definition of cell subpopulations. Moving forward, we will deepen our research at the molecular level, aiming to characterize and understand the fundamental traits of these different cell subpopulations. By delving into their molecular characteristics, we hope to reveal the intrinsic nature of cell subpopulations, facilitating more accurate population delineation, thereby aiding clinical practice in obtaining higher purity, specific function cell subpopulations for more refined, stable, and targeted treatment plans for patients (Figure 1B).

### 3.2. Harnessing scRNA-seq to dissect the molecular features of MSCs

#### 3.2.1. MSC developmental trajectory typing

Various research groups have distinct understandings and discoveries regarding the developmental trajectories of MSCs. For instance, Wang *et al.*, by reconstructing developmental trajectories across donors for MSCs from multiple tissue sources, discovered that UC-MSCs display a higher degree of donor variability than other sources. By evaluating the expression trajectories of ECM related genes across different tissues, they observed that changes in the ECM could promote the heterogeneity of UC-MSCs, thereby affecting the stemness of UC-MSCs 30. Ma *et al.* analyzed the developmental trajectories of MSCs from the perspectives of intercellular heterogeneity and the adaptability of cellular responses within the organism. They found that microenvironments rich in cytokines and growth factors play a pivotal role, potentially triggering biological defense responses, thus playing a crucial role in maintaining cellular developmental potential, plasticity, and a wide range of cellular functions 34. To study the developmental trajectories of different MSC subpopulations and determine the developmental starting points, Xie *et al.*, through trajectory branching analysis, identified that the stemness subpopulation irreversibly differentiates into functional subpopulations or proliferative subpopulations. Furthermore, by analyzing the proportions of each subpopulation in MSCs across different passages, they discovered that the proportions of functional subpopulations and senescent MSCs increase with passage, indicating that the functionality of MSCs might also primarily depend on the proportion of functional subpopulations assessed at the time point 16. Chen *et al.*'s study also focused on the developmental trajectories of MSC subpopulations, finding that subpopulations with high proliferation capabilities can transform into other subpopulation cells. Moreover, they noted that biologically functional MSCs could highly express immune-related and angiogenesis-promoting genes, while niche-supporting MSCs are the central subpopulations in MSCs, coordinating various aspects of MSC functionality 33. Once removed from their niche, the primitive gene expression activities related to stem cell niche support may be lost 35. Overall, these studies, by delving into the developmental trajectories of MSCs, have unveiled the dynamic changes in MSCs during differentiation. This has the potential to identify key factors affecting MSC differentiation, providing crucial insights for controlling cell fate decisions.

Having studied the developmental trajectories of MSCs and understood the processes and mechanisms of cellular differentiation, we can further delve into the issue of cell state classification. Research on the developmental trajectories of MSCs provides a wealth of information and theoretical basis for cell state classification, which deepens and extends the research on MSC developmental trajectories. Cell state classification mainly involves categorizing and naming the states of cells at different developmental stages, which is crucial for understanding cellular functions and guiding cell therapy. Within the developmental trajectory of MSCs, we observe various cell states, all of which can serve as references for cell state classification. A deeper understanding and classification of these cell states not only helps us further comprehend the biological characteristics and functions of MSCs but also aids in optimizing the clinical application of MSCs. This involves selecting cell states more suitable for treating specific diseases or improving treatment outcomes by regulating cell states. Therefore, we will next explore the topic of cell state classification in more detail (Figure 1C).

#### 3.2.2. MSC cell state typing

To explore the significance of different functional cell states among subpopulations, Wolock *et al.* predicted and validated transcription factors that control stromal cell differentiation. This revealed various differentiation states of mature stromal cells and deepened our understanding of the complexity of cell states within the BM microenvironment 32. Huang *et al.* discovered an inverse relationship between cell cycle gene modules and immune-related molecular modules, which is also reflected in age-related secretory phenotypes. Utilizing a newly developed cell cycle scoring algorithm, they found that most UC-MSC subpopulations predominantly occupy the G2/M phase of the cell cycle, indicating that the regulation of UC-MSCs' predominant cellular state is governed by the cell cycle process, irrespective of their expression of inflammatory cytokines 26. Wang and his team, by studying inter-tissue transcriptome regulons and protein interactions, identified several prominently activated regulators, reflecting the heterogeneity of cell states within tissues. They also found that characteristic genes of each subpopulation are clustered within the same functional terms, suggesting that these genes might exhibit similar cellular state functions when co-expressed 30.

Cell developmental typing and cell state classification are two critical research areas in cell biology. Both types of classification research can help us identify cell subpopulations with special functions, providing important information for applications such as disease treatment and regenerative medicine. Next, we will delve into the details of MSC special functional subpopulation classification (Figure 1C).

#### 3.2.3. MSC specific functional subpopulation typing

The successful isolation of effective functional MSC subpopulations plays a crucial role in the construction of tissues or organs *in vitro*, the development of novel drug carriers, and the treatment of various clinical diseases. Xie *et al.*, utilizing scRNA-seq, identified the characteristic phenotype CMKLR1 within the functional subpopulations. Through further studies involving ALP staining, ORO staining, and *in vivo* models, they discovered that the isolated CMKLR1-MSC functional subpopulation exhibits superior immunoregulatory and osteogenic differentiation capacities, albeit with lower adipogenic differentiation and proliferation potentials 33. Notably, Chen *et al.* predicted two previously unreported transcription factors, ELK3 and RREB1, through TF prediction analysis. These factors serve as vital drivers for the biofunctional-type_MSCs involved in wound repair. They also purified this subpopulation based on cell-surface marker genes and representative pro-angiogenic genes (S100A9CD29CD142 cells correspond to biofunctional type_MSCs) to confirm their potency in wound healing. *In vitro* results demonstrated that S100A9CD29CD142 MSCs positively influence keratinocytes/fibroblasts/endothelial cells by promoting cell proliferation and migration, essential for wound healing. Furthermore, in a zebrafish skin injury model, wounds treated with this subpopulation healed faster and exhibited an accelerated re-epithelialization process, indicating superior quality of wound healing compared to treatments with unclassified MSCs 33. The advancements hold immense significance in terms of the standardization of cellular products for clinical translation and development of cell-based therapies.

Furthermore, in order to better understand the spatial organization and global gene expression profiles of cell types in the Wharton's Jelly, Chen *et al.* utilized ST analysis to investigate four distinct regions of the same UC. They found a higher proportion of biofunctional-type_MSCs in the fetal population compared to the maternal population, suggesting that biofunctional-type_MSCs from fetal segments may be a preferred source for wound repair. The study also revealed that different regions of the same donor UC exhibit distinct spatial interactions among cell types, and specific interaction patterns exist between different cell types in UC regions among different donors, further confirming the existence of spatial cellular heterogeneity. Additionally, it was discovered that anti-aging-related gene expression profiles in the niche-supporting_MSCs and biofunctional-type_MSCs are similar, and they exhibit strong co-localization in space. The combined application of these two subpopulations may lead to surprising effects. In summary, this study elucidates the relationship between cellular subpopulation functionality and spatial distribution, and provides innovative therapeutic approaches for tissue regeneration using specific subpopulations alone or in combination, which holds particular significance for assessing treatment responses in diseases 33 (Figure 1C).

By delving into the heterogeneity of MSCs, we can not only select high-quality cell subpopulations with specific functions, thereby enhancing treatment stability and optimizing the benefits of cell therapy but also guide our efforts through engineering approaches to further improve treatment outcomes. Engineering modifications are primarily of two types: first, employing genetic engineering to enhance the innate capabilities of MSCs and endow them with new functions; second, adopting tissue engineering methods from a materials science perspective to improve the therapeutic effects of MSCs.

## 4. Enhancing the efficacy of POF treatment through genetic engineering of MSCs

MSCs have shown significant efficacy in treating premature ovarian insufficiency by improving folliculogenesis, reducing granulosa cell apoptosis, promoting angiogenesis, increasing pregnancy rates, and regulating hormonal balance 36. Genetic engineering offers unique advantages to enhance the innate capabilities of MSCs, such as increasing their proliferation and differentiation capacity, improving migration and homing ability, enhancing adhesion, delaying aging, and boosting survival rates. Moreover, genetic engineering can bestow new functions on MSCs, including precise regulation of therapeutic switches to enhance targeting and reduce potential side effects, and serving as carriers for delivering various molecules to augment therapeutic outcomes. Given the current limited research on genetically engineered MSCs for ovarian function restoration, this review summarizes the relevant studies and explores common genetic engineering strategies to enhance MSC functionality for POF treatment, providing more specific references for future research.

### 4.1. Enhancing the innate abilities of MSCs

#### 4.1.1. Boosting proliferation and differentiation of MSCs

In the research on treating POF, genetic engineering is considered a highly promising strategy, aiming to repair and regenerate ovaries by enhancing MSC proliferation and differentiation potential. This strategy hinges on regulating relevant cytokines through overexpression of growth factors and interleukins, as well as the knockout or overexpression of key transcription factors. These interventions manipulate the intrinsic mechanisms of cells, altering their biological properties to improve their efficacy in ovarian regeneration. For example, TGF-β1 plays a crucial role in cell growth, differentiation, immunosuppression, and repair after injury 37. Transplanting human UC-MSCs (hUC-MSCs) into a POF rat model significantly reduced TGF-β1 expression, and the use of its inhibitors further confirmed that hUC-MSCs enhance MSC proliferation and differentiation via the TGF-β1/Smad3 signaling pathway, thereby inhibiting the expression of fibrosis markers (α-SMA and Collagen III) and significantly improving ovarian function 38. Hepatocyte growth factor (HGF) is a vascular regulator located in ovarian cells that modulates hormone levels and granulosa cell proliferation. The Wnt signaling pathway, activated by HGF, positively regulates MSC proliferation and differentiation 39. Park and colleagues demonstrated that HGF secreted by placental-derived MSCs (PD-MSCs), through Wnt pathway activation, increased MSC proliferation and differentiation, improving ovarian function in a rat model of partial ovariectomy by remodeling ovarian vasculature, promoting follicle development, luteinization, and steroidogenesis 40.

**Figure 2:**
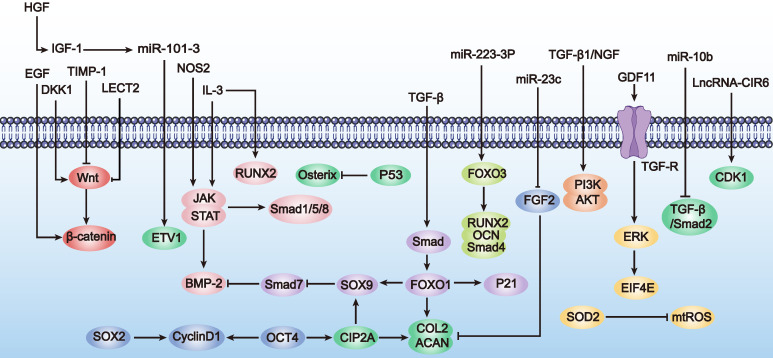
** Diagram illustrating factors that promote MSCs proliferation and differentiation (as outlined in Table 1).** The figure highlights key substances and their effects on various downstream signaling pathways and molecules. Arrows (→) indicate activation or upregulation, while lines (⊥) indicate inhibition. Key pathways such as IGF-1 secretion and TGF-β signaling are depicted.

In conclusion, enhancing MSC proliferation and differentiation through genetic engineering is a promising new strategy for treating POF. Although this research field is still in its infancy and studies are limited, these preliminary results show that optimizing MSCs via genetic modification brings new hope for POF treatment. Therefore, we also summarize the applications of genetically engineered MSCs in enhancing proliferation and differentiation in other disease types (Table 1 and Figure 2). These studies provide valuable references for further exploration and optimization of MSC applications in POF treatment.

#### 4.1.2. Enhancing migration and homing abilities of MSCs

MSCs possess remarkable migratory abilities, enabling them to traverse endothelial barriers and reach sites of tissue injury and inflammation 41. Studies indicate that in the context of POF, MSC migration and homing behaviors are regulated by various chemical signals, including chemokines and growth factors. Among these, stromal cell-derived factor-1 (SDF-1) and its receptor CXC chemokine receptor 4 (CXCR4) play crucial roles in MSC migration and homing 7. SDF-1 is highly expressed in damaged tissues, while CXCR4 is predominantly expressed in BM-MSCs, interacting to control cell proliferation, differentiation, and migration, thereby promoting wound repair and regeneration. This interaction is essential in processes such as germ cell development, angiogenesis, and muscle regeneration 42. To investigate the role of the SDF-1/CXCR4 axis in hAD-MSC transplantation in POF mice, Li *et al.* used cyclophosphamide to establish a POF rat model and transplanted hAD-MSCs into the ovaries. The results showed increased SDF-1/CXCR4 expression in the ovaries post-transplantation, activating the PI3K/AKT signaling pathway and promoting hAD-MSC homing to the POF ovaries 43. In another study, researchers treated mouse ovaries with hUC-MSC secretome (hUC-MSC-sec) and a PBS control. Compared to the control group, hUC-MSC-sec-treated mice exhibited significantly larger ovaries and increased follicle activation. Mechanistic exploration revealed that hUC-MSC-sec treatment enhanced ovarian AKT phosphorylation and activated the SDF-1/CXCR4 axis via HGF secretion, promoting follicle activation and enhancing MSC migration and homing to the POF ovaries 44. Additionally, overexpressing autocrine signals through aquaporin-1 and CXCR4 also promoted MSC migration to injured sites by activating the PI3K/AKT and MAPK/Erk signaling pathways 45. Therefore, improving MSC migration and homing abilities is beneficial for POF treatment.

**Figure 3:**
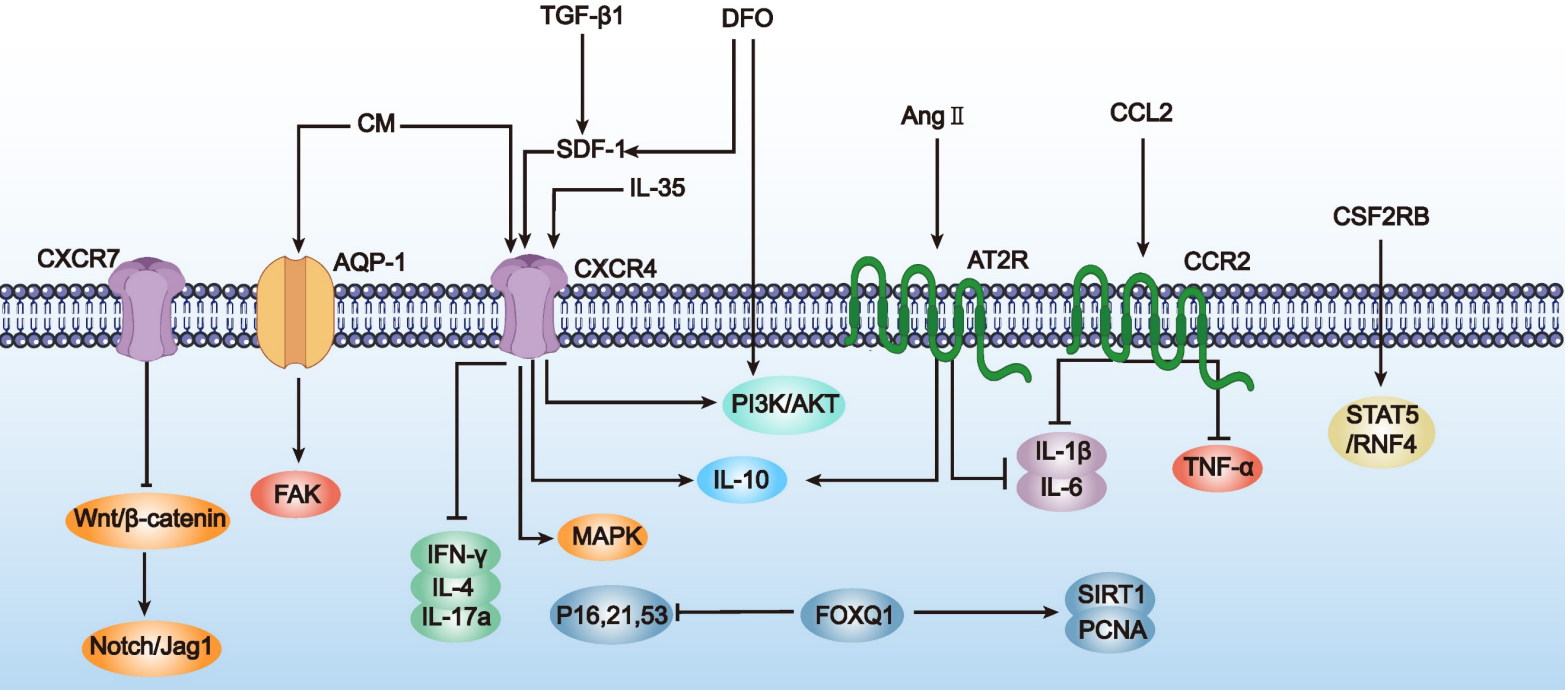
** Schematic representation of the strategies to enhance MSC migration and homing abilities (as outlined in Table 2)**. This figure focuses on the activation (→) and inhibition (⊥) of pathways such as SDF-1/CXCR4 and PI3K/AKT, demonstrating their roles in facilitating MSC movement towards injury sites.

In summary, preliminary studies suggest that enhancing MSC homing abilities through genetic modification can effectively improve ovarian function in POF mice. Although this research field is still in its infancy and studies are limited, these initial results demonstrate the potential of genetic modification techniques to enhance MSC homing for POF treatment. Additionally, we summarize the research directions for genetically engineered MSCs to improve homing abilities in other disease types (Table 2 and Figure 3), which may provide more specific design references for MSC-based POF therapy. Future research needs to explore the mechanisms by which modified MSCs affect ovarian function more deeply and assess their long-term safety and efficacy.

#### 4.1.3. Enhancing adhesiveness

In the treatment of POF and its complications, MSC adhesion plays a critical role. During the treatment of injury or disease, MSC adhesion enables them to localize and migrate to damaged tissues or organs. For instance, post-cell transplantation, MSCs are prone to apoptosis or necrosis due to the loss of adhesion to the matrix, leading to low survival rates. However, MSC adhesion aids their survival and functional maintenance in the recipient tissues after transplantation 46. Thus, enhancing cell adhesion can improve post-transplant survival rates, thereby increasing the clinical success of MSC applications.

**Figure 4:**
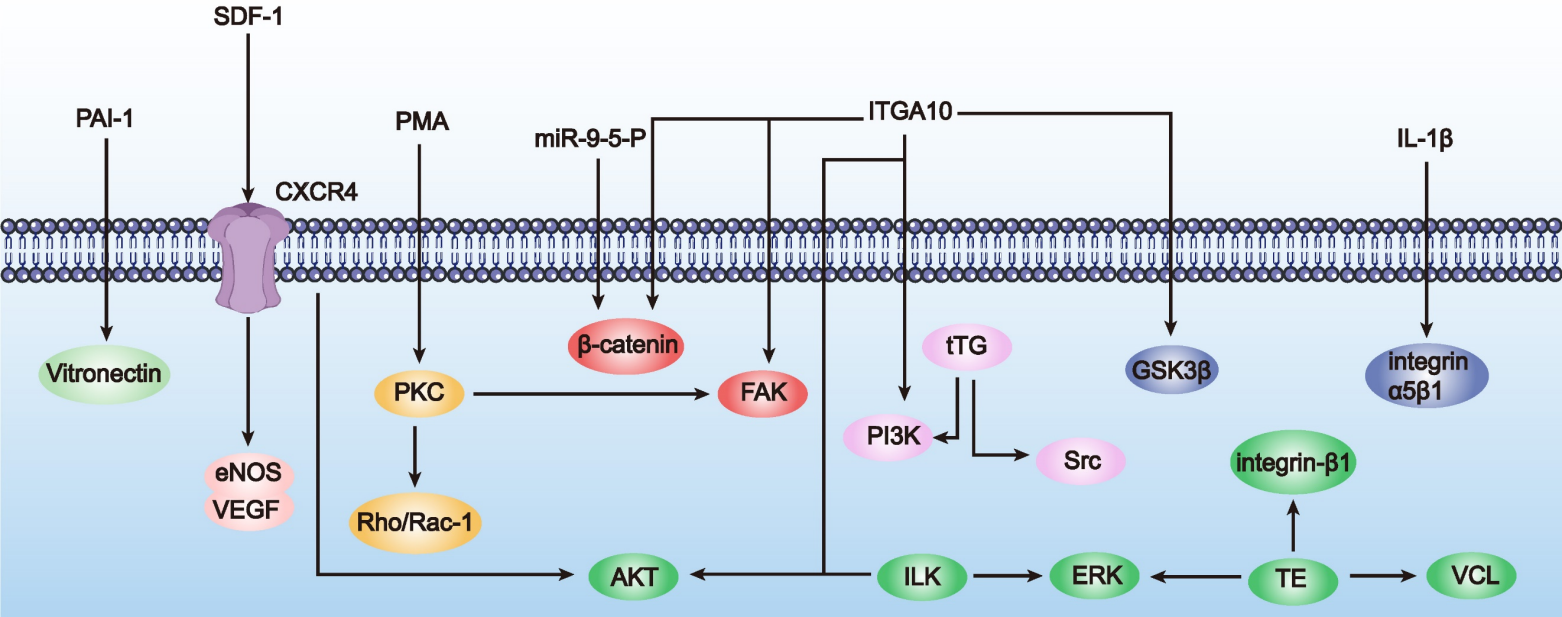
** Visual representation of mechanisms enhancing MSC adhesiveness (as outlined in Table 3).** The figure illustrates the regulatory (→) and inhibitory (⊥) effects of various agents on key signaling pathways, including FAK and Rho/Rac-1, which are crucial for cell adhesion.

Although research on enhancing MSC adhesion through genetic engineering for POF treatment is relatively scarce, there are numerous successful cases in other diseases, such as osteoporosis and osteoarthritis, which are complications of POF. Next, we will introduce successful genetic engineering methods to enhance MSC adhesion in other diseases, hoping to provide insights for POF treatment (Table 3 and Figure 4). Mainstream genetic engineering strategies to enhance MSC adhesion can be considered from two aspects: surface modification and gene regulation.

**a) Surface modification.** Surface modification involves introducing specific molecules such as collagen or fibronectin to the cell surface to increase adhesion to the matrix. This can be achieved through chemical modification or genetic engineering. For instance, overexpression of tissue transglutaminase (tTG) or integrin-related proteins (such as focal adhesion kinase (FAK) and integrin-linked kinase (ILK)) can enhance MSC adhesion, expansion, and migration capabilities 46. IL-1β enhances hMSC adhesion by increasing the availability and clustering of integrin α5β1 on the cell membrane, providing new insights into integrin clustering during inflammation and a rational basis for improving hMSC engraftment 47.

**b) Gene regulation.** Gene regulation involves modulating the expression of adhesion-related genes to influence the regulation of adhesion signaling pathways and related proteins, thereby enhancing cell adhesion. For instance, inhibiting the expression of prolyl hydroxylase domain protein 2 (PHD2) in BM-MSCs can increase the stability of hypoxia-inducible factor-1α (HIF-1α), enhancing cell viability. BM-MSCs protect ischemic myocardial cells by secreting insulin-like growth factor 1 (IGF-1) and other protective factors, thereby enhancing adhesion between cells and myocardium 48. Therefore, engineering to modulate adhesion-related gene expression can enhance cell adhesion, aiding their survival and functional maintenance in recipient tissues, providing new strategies for improving MSC function and implant integration 49.

Although these strategies and techniques are mainly applied to other disease treatments, they offer potential methods and directions for enhancing MSC adhesion in POF treatment. Future experimental validation and clinical trials could apply these genetic engineering approaches to POF treatment, improving therapeutic outcomes and patient quality of life.

#### 4.1.4. Decelerating premature senescence in MSCs

**Figure 5:**
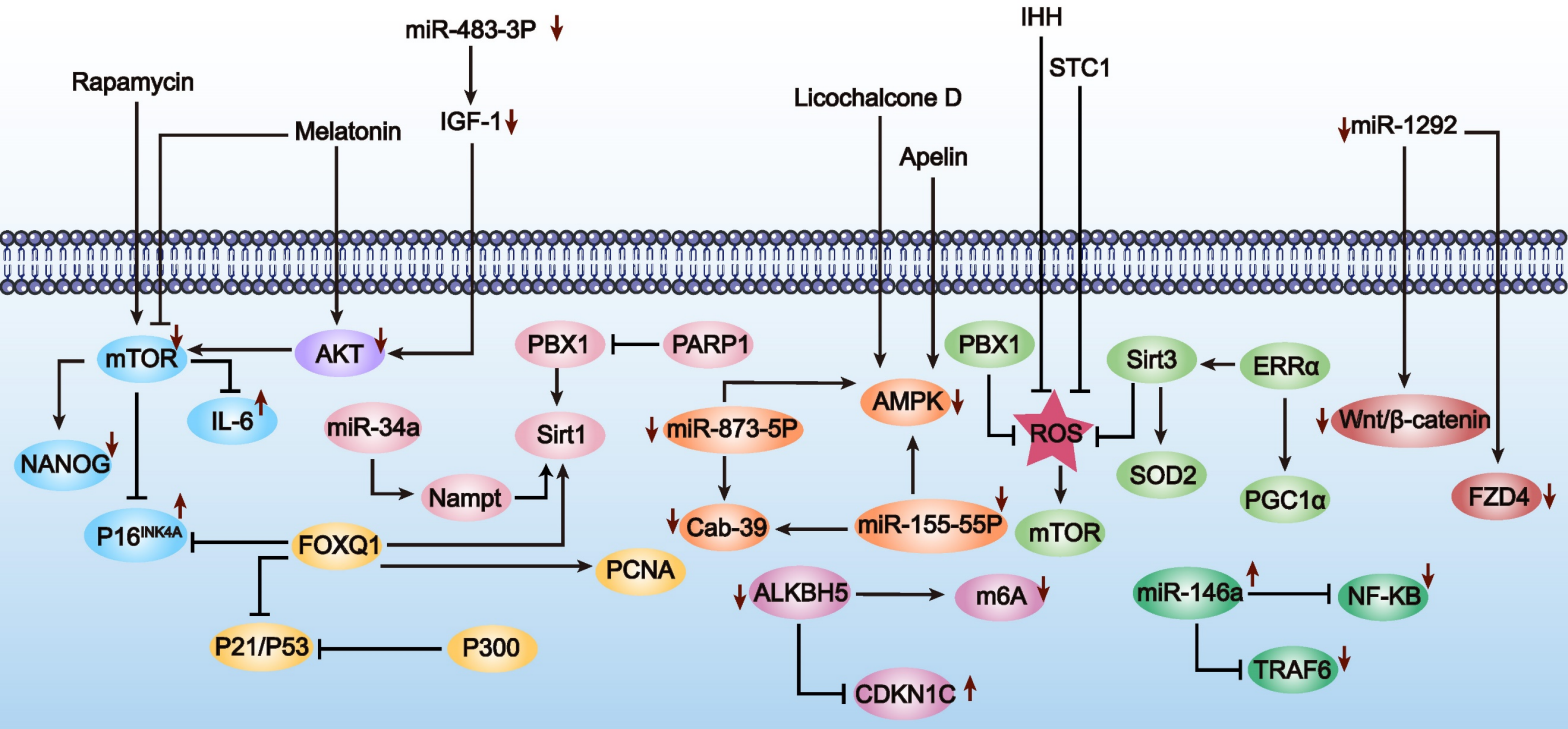
** Diagram showing interventions aimed at decelerating premature senescence in MSCs (as outlined in Table 4).** The figure highlights the influence of mTOR inhibition (⊥) and AMPK activation (→), among other mechanisms, on slowing down cellular aging processes. Furthermore, the upward arrow (↑) signifies that the upregulation of this substance postpones cell senescence, and the downward arrow (↓) indicates that the downregulation of this substance has a similar effect.

Over the past few decades, MSCs have been widely used in anti-aging therapies due to their easy accessibility, simple isolation procedures, robust self-renewal capacity, and multipotent differentiation potential 50. However, with increasing age or prolonged culture time *in vitro*, the functionality of MSCs gradually declines, which limits their application in the treatment of POF 51. The aging of MSCs is characterized by genetic material damage, imbalanced regulation of non-coding RNAs, loss of protein stability, disruption of intracellular signaling pathways, and mitochondrial dysfunction. Therefore, addressing the issue of premature MSC aging is crucial to maintaining their optimal immunomodulatory capabilities, as aging disrupts their essential biological activities 52. Given the limited data on genetically engineering MSCs to mitigate premature aging for POF treatment, we summarize the key characteristics of MSC aging and explore genetic engineering approaches used to delay MSC aging in other diseases, hoping to provide insights for POF therapy (Table 4 and Figure 5).

**a) Repairing genetic material damage.** Genetic material damage involves genomic instability, telomere shortening, and epigenetic changes 52, with DNA damage being a primary cause of stem cell aging. Studies have shown that ROS can induce DNA damage and affect the DNA damage response 53. Overexpression of pre-B-cell leukemia homeobox 1 (PBX1) via lentiviral vectors can mitigate ROS-mediated DNA damage and thereby attenuate hair follicle-derived mesenchymal stem cells (HF-MSCs) aging 54.

**b) Non-coding RNA regulation.** Non-coding RNAs play diverse roles in various cellular processes, including protein translation and gene expression regulation. Utilizing them to delay MSC aging is a viable approach 55. Recent studies indicate that miR-34a is closely associated with MSC aging. Lentiviral transfection of miR-34a induced age-related MSC aging, which could be alleviated by targeting Nampt via the NAD-Sirt1 pathway 56. miRNA, differentially expressed in MSCs and regulated by SASP cytokines, holds significant potential for improving MSC aging by eliminating harmful senescent cells and their inflammatory secretions 57.

**c) Intracellular signaling pathways.** Signal transduction refers to a series of molecular processes within cells that transmit and translate information, adjusting physiological functions and adaptive responses to internal and external environmental changes. MSC aging is also regulated through signaling pathways, including mTOR, AMPK, IGF1, SIRT1, and P53. We discuss how modulating these pathways can delay MSC aging, with implications for POF treatment. For instance, Yun *et al.* pretreated MSCs with melatonin *in vitro*, successfully inhibiting mTOR and AMPK signaling pathways while activating the PI3K/AKT pathway. This effectively inhibited p-Cresol-induced ROS accumulation and autophagy, preventing MSC aging induced by the uremic toxin p-Cresol 58. The IGF1 signaling pathway, also known as the growth hormone axis, is a significant focus in MSC aging research 59. Knockdown of miR-483-3p expression can delay the aging of hAD-MSCs by upregulating the IGF1 signaling pathway 60. Moreover, downregulating the P53 pathway also plays a positive role in delaying MSC aging. In hUC-MSCs, knockdown of E1A binding protein (p300) upregulated p53 and p21 expression, promoting MSC aging and inhibiting growth, indicating the crucial role of the p53 pathway in combating MSC aging 61.

#### 4.1.5. Improving MSC survival rates

**Figure 6:**
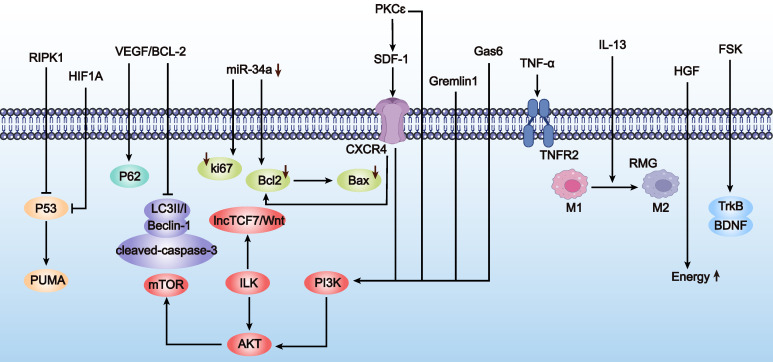
** Schematic representation of strategies to improve MSC survival rates (as outlined in Table 5).** The figure depicts the upregulation (→) and inhibition (⊥) of survival-related pathways such as PI3K/Akt and SDF-1/CXCR4, underlining the impact of various factors on enhancing MSC viability. Furthermore, the upward arrow (↑) signifies that the upregulation of this substance improve MSC survival rates, and the downward arrow (↓) indicates that the downregulation of this substance has the same effect.

Enhancing the survival rate of MSCs in the treatment of POF is crucial. This advancement could potentially allow therapeutic effects to be achieved with fewer MSCs, thereby reducing treatment costs. Genetic engineering of MSCs has been shown to effectively improve their survival in the POF environment. However, due to limited studies, we have also comprehensively summarized strategies from other diseases to enhance survival, providing insights for the treatment of POF (Table 5 and Figure 6). These modifications mainly focus on the following aspects:

**a) Signal pathway regulation.** Key pathways, such as the P53 signaling pathway, are critical in regulating MSC survival. miR-34a is a downstream target of the P53 pathway; P53 activation induces miR-34a expression both *in vitro* and *in vivo*. Using miR-34a inhibitors to transfect MSCs and inducing oxidative stress with H_2_O_2_, studies have shown that downregulating miR-34a levels can increase the expression of Bcl-2, survivin, and Ki67, thereby enhancing MSC survival under oxidative stress conditions 62.

**b) Protein kinase regulation.** Overexpression of ILK in iPSC-MSC-EVs applied to granulosa cells can reduce apoptosis, enhance cell proliferation, and improve granulosa cell viability via the ILK-PI3K/AKT pathway 63. ILK is considered vital for promoting cell survival, and genetic modification of ILK is seen as a promising approach to enhance MSC survival rates. Studies have shown that ILK-overexpressing MSCs exhibit increased survival and promote angiogenesis via upregulating AKT and mTOR pathways after transplantation in acute myocardial infarction models 64. Additionally, ILK overexpression under hypoxic conditions can boost MSC survival and self-renewal abilities, as shown in studies where elevated IL-6 levels activated the JAK2/STAT3 signaling pathway and significantly upregulated lncRNA 65.

**c) Growth factor regulation.** VEGF is a primary regulator of ovarian angiogenesis, and insufficient vascular supply can limit follicular growth and lead to follicular atresia 66. Engineering MSCs to upregulate VEGF expression, such as through P311 gene modification, can promote angiogenesis and wound healing 67. Another study found that co-overexpressing VEGF and Bcl-2 in MSCs significantly reduced apoptosis and improved survival under oxygen-glucose deprivation conditions 68. Although specific studies on VEGF in POF are lacking, PD-MSCs have shown that VEGF pathway activation of PI3K/AKT/mTOR and GSK3β/β-catenin pathways promotes angiogenesis and follicular development to restore ovarian function 66.

**d) Chemokine regulation.** SDF-1 is a chemokine crucial for stem cell migration, acting as a homing factor for stem cells. It exerts its effects by binding to CXCR4 69. Beyond cell migration, SDF-1 and CXCR4 binding activates AKT and Erk pathways, enhancing the survival and proliferation of bone marrow MSCs while regulating apoptosis.

**e) Other regulatory methods.** Other modifications to enhance MSC survival include overexpressing Gremlin1 70, upregulating tumor necrosis factor receptor 2 (TNFR2) 71, and genetically modifying MSCs to overexpress Gas6 72 and IL-13 73. Overexpression of hypoxia-inducible factor 1α (HIF1A) 74 and upregulation of BDNF receptor (TrkB) expression 75 are also effective strategies. For instance, genetic modification to overexpress Gas6 significantly reduced apoptosis and increased MSC survival both *in vitro* and *in vivo* post-transplantation, improving left ventricular function and reducing myocardial infarction area 72.

In summary, enhancing MSC survival through signal pathway regulation, growth factor modulation, chemokine regulation, and other methods can reduce treatment costs, expand clinical applications, and improve research efficiency. Genetic engineering strategies to boost MSC survival in POF treatment warrant in-depth investigation. These strategies not only reduce the number of grafts required but also extend the survival time of MSCs in the POF microenvironment, providing prolonged and potent therapeutic effects. This has significant implications for promoting MSC clinical applications in POF treatment. Furthermore, endowing MSCs with new biological functions to expand their potential in POF therapy merits further exploration. Next, we will detail how genetic engineering can imbue MSCs with new functionalities.

### 4.2. Beyond innate function

#### 4.2.1. Activation of MSCs by small molecule compounds to regulate disease progression

By using small molecule compounds to activate MSCs, we can achieve precise control over the activation timing and location of MSCs in the treatment of POF. This approach reduces MSC consumption at non-target sites, enhances efficacy at target sites, and minimizes potential side effects, thereby improving treatment safety. Cell activation (also known as licensing or preconditioning) is an immunological concept applied in stem cell therapy 76. Common activation methods include: a) Using pro-inflammatory cytokines or growth factors: For example, IL-1-activated MSCs increased G-CSF expression via IL-1R1, reducing inflammatory mediator secretion in LPS-activated microglia and steering human MSCs towards an anti-inflammatory and pro-trophic phenotype *in vitro* 77. b) Using hypoxia: Hypoxia (2-2.5% O_2_) induced P-MSCs to secrete insulin, upregulate glucose transporters and adhesion molecules, exhibit increased angiogenic potential, and promote wound healing 78. c) Using drugs and chemical agents: For example, UC-MSCs activated with VPA+S1P (valproic acid + sphingosine-1-phosphate) showed upregulation of gene subsets associated with stem cell migration and anti-inflammatory responses 79.

Using small molecules to activate MSCs not only increases their local concentration at target sites, maximizing their repair and regenerative effects, but also avoids systemic side effects that might result from excessive activation. Several factors have been identified as switches for MSC therapy in neurodegenerative diseases. For example, Nurown involves MSCs secreting high levels of neurotrophic factors, differentiated from bone marrow-derived MSCs. These factors include GDNF, BDNF, VEGF, and HGF, induced by a proprietary medium formulation. MSCs secreting neurotrophic factors have been shown to be safe for repeated transplantation 80. In treating neurodegenerative diseases such as ALS, clinical trials by Panayiota Petrou *et al.* have shown that patients receiving intrathecal or intramuscular plus intrathecal transplantation had at least a 25% improvement in progression slope over 6 months compared to controls 81.

**Figure 7:**
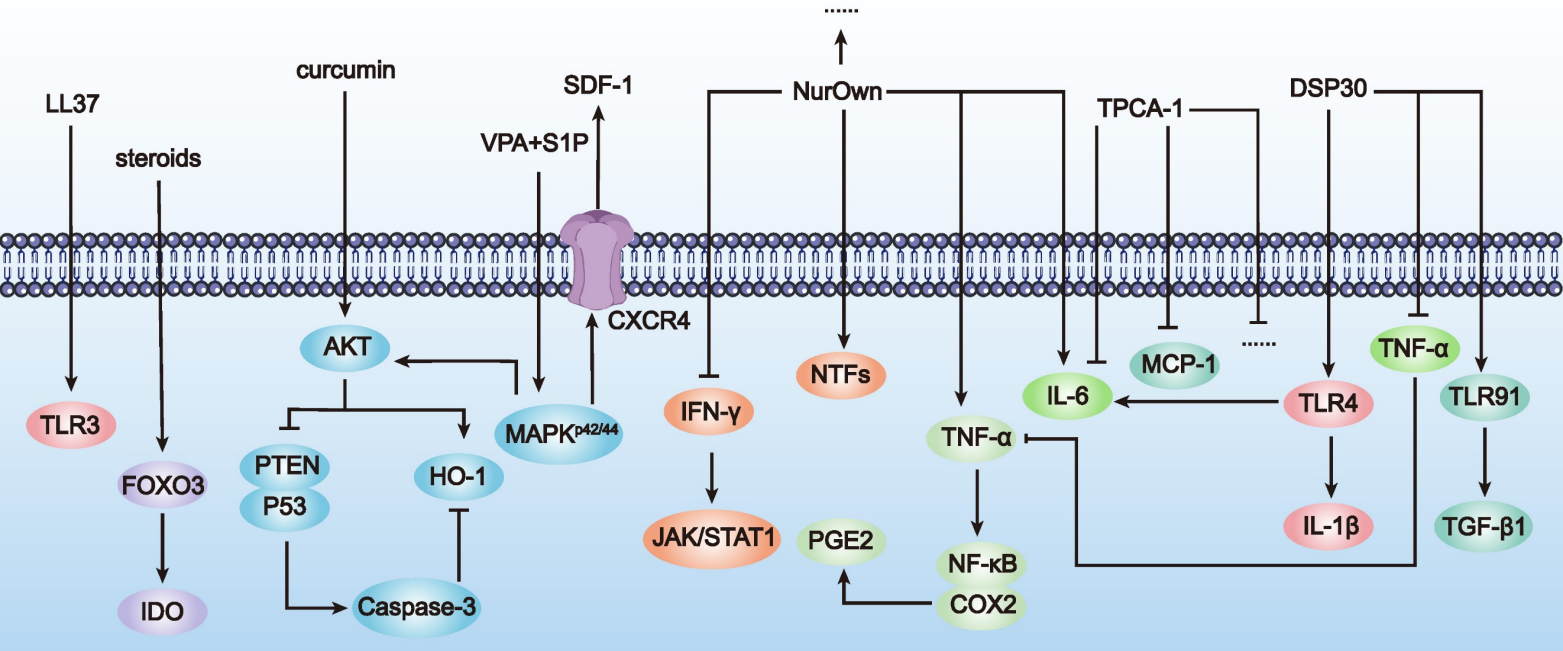
** Illustration of MSCs acting as a therapeutic switch (as outlined in Table 6).** The figure showcases how different agents, such as budesonide and curcumin, modulate MSC functions by activating (→) or inhibiting (⊥) specific pathways and molecular targets, thereby influencing therapeutic outcomes.

In summary, with the ongoing discovery of small molecules like neurotrophic factors and chemokines secreted by MSCs, there will be more opportunities to use these compounds as switches to control MSC activation in POF treatment. This will enable precise control over the activation timing and location of MSCs *in vivo*, reduce their consumption, enhance therapeutic efficacy, and lower toxic side effects. Achieving this would represent a significant breakthrough in improving the efficacy and safety of MSC-based therapies for POF treatment (Table 6 and Figure 7).

#### 4.2.2. MSCs as carriers to enhance therapeutic efficacy

Through genetic engineering, we can modify not only the biological properties of MSCs themselves but also use them as carriers to deliver exogenous therapeutic genes or proteins, thereby enhancing the treatment of POF.

The application of genetic engineering allows specific genes to be inserted into the DNA of MSCs, enabling them to localize to targeted therapeutic areas or secrete specific therapeutic molecules, thereby further enhancing therapeutic efficacy. For example, engineering MSCs to express glial cell line-derived neurotrophic factor (GDNF) has shown greater efficacy than direct infusion of the neurotrophic factor, offering new hope for Parkinson's disease 82. Genetically modified MSCs that overexpress brain-derived neurotrophic factor (BDNF) have been shown to improve symptoms in a mouse model of Huntington's disease. These engineered MSCs significantly enhance motor function, reduce neurodegeneration and inflammation, and improve cognitive function compared to unmodified MSCs 83.

Overall, genetic engineering of MSCs can transform them into highly effective therapeutic carriers, enhancing treatment outcomes. Genetic modifications not only regulate and optimize the biological properties of MSCs but also enable them to serve as efficient carriers for delivering various therapeutic genes and molecules. This will undoubtedly expand and enhance the application of MSCs in the treatment of POF. MSCs represent an ideal biological carrier, poised to significantly improve the efficacy of gene and molecular therapies in ovarian pathologies. This represents a critical direction for future MSC therapeutic research (Table 7).

## 5. Tissue engineering

The application of genetic engineering in the field of MSCs has achieved significant breakthroughs. Precise genetic manipulation allows us to not only enhance the inherent functions of stem cells but also bestow them with novel capabilities. This not only broadens the application scope of stem cells in regenerative medicine but also presents MSCs with greater therapeutic potential. However, genetic engineering alone does not resolve all issues. On one hand, the effective survival and functionality of genetically modified MSCs *in vivo* are influenced by various factors, including the *in vivo* microenvironment. On the other hand, the transplantation and application of MSCs require careful consideration of their compatibility with the host. Therefore, a broader perspective is necessary, specifically the tissue engineering modification of MSCs.

Tissue engineering, by constructing suitable biomaterial platforms, can effectively enhance the survival and functional performance of MSCs *in vivo*, thereby improving therapeutic outcomes. Next, we will delve into how tissue engineering can modify MSCs from a materials science perspective to enhance their clinical efficacy.

### 5.1. Scaffold-free approaches

Scaffold-free approaches, leveraging the inherent capabilities of cells to mimic developmental processes for the formation of *in vitro* organotypic 3D tissue substitutes without reliance on scaffolds, present a significant advancement in therapeutic potential through improved implantation efficiency 84. This methodology has found particular application in ovarian research, focusing on self-assembled spheroids such as microgels, spheroids, and nanoparticles. These scaffold-free cultures offer numerous advantages in enhancing ovarian function. For instance, Krotz *et al.* successfully created a 3D artificial human ovary by seeding Theca and granulosa cells isolated from the follicles of women of reproductive age into micro-molded agarose gels made from polydimethylsiloxane casting. This complex micro-tissue maintained viability for a week, with Theca cells fully encapsulating granulosa spheroids or Cumulus granulosa-oocyte complexes without matrix invasion or damage after 72 hours of artificial ovary construction. Unlike using alginate or collagen scaffolds, Theca cells in this construct continued to produce hormones throughout the oocyte development process, suggesting that artificial human ovaries might more effectively mature primordial oocytes into fertilizable mid-stage II oocytes 85. Yoon *et al.* employed a microchannel network hydrogel containing cellular spheroids (vascularized hydrogel with ovarian spheroids, VHOS), implanted into the ischemic hind limbs of rats undergoing ovarian removal. This approach significantly promoted hormone release and restoration of endocrine function, leading to complete regeneration of the endometrium. VHOS implantation effectively suppressed the side effects observed with synthetic hormone therapy, such as tissue overgrowth, proliferation, cancer progression, and deep vein thrombosis, reducing these side effects to normal levels. Simultaneously, it also effectively prevented typical sequelae of menopause, such as increased adiposity and induction of osteoporosis. 86. Kim *et al.* compared the therapeutic effects of PD-MSCs cultured traditionally in two-dimensional (2D, naive) systems versus three-dimensional (3D, spheroid) systems. They discovered that, compared to 2D cultures, spheroid-cultured PD-MSCs extended ovarian function, generated more follicles, and the estradiol level in the spheroid group was significantly higher than that in the Naive group at 2 weeks.

Furthermore, there was an increase in the expression of folliculogenesis-related genes like Nanos3, Nobox, and Lhx8 at both one and two weeks, suggesting that spheroid-cultured PD-MSCs could enhance therapeutic potential by improving implantation efficiency 87.

However, the use of nanoparticles (NPs) in enhancing ovarian function presents a double-edged sword. While encapsulating drugs in NPs has shown promise due to lower cytotoxicity and higher cellular uptake, effectively lowering serum levels of LH, prolactin, testosterone, and insulin, NPs could adversely affect female reproductive health by altering normal ovarian structure and sex hormonal levels 88, 89. Studies indicate that exposure to NPs can disrupt mammalian reproductive functions by changing steroid hormone secretion levels. Furthermore, excessive dosages of quantum dots can interfere with oocyte maturation, reduce hormone receptor miRNA levels, and diminish the potential for *in vitro* fertilization 90.

**Figure 8:**
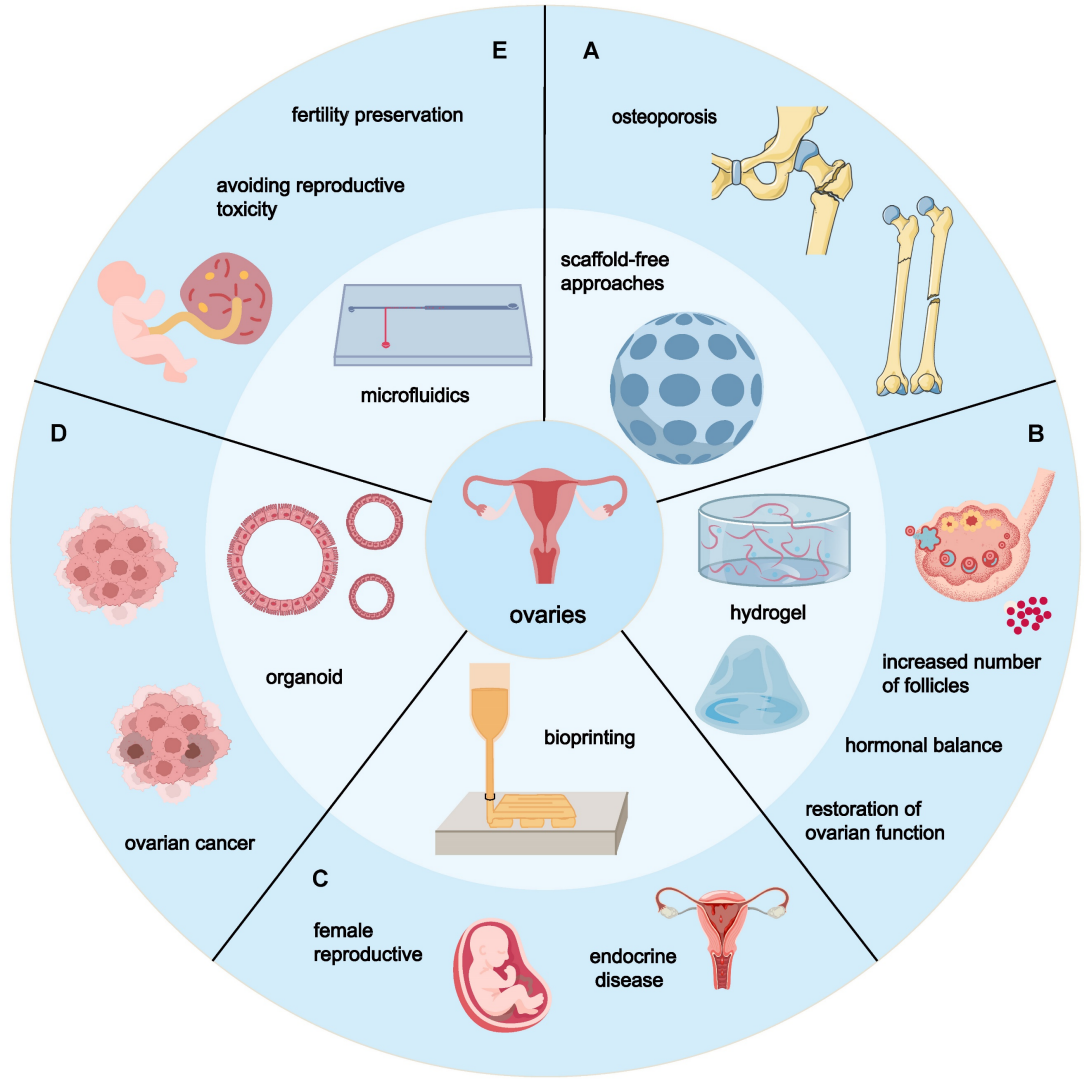
** Tissue engineering of MSCs with different materials and their main targets for the complications of POF.** (A) The use of unstructured cell culture can effectively prevent postmenopausal sequelae, including increased fat mass and induced osteoporosis. (B) Hydrogel engineering can increase the number and density of follicles in mice, restore hormonal balance, and restore ovarian function. (C) Bioprinting has broad clinical prospects in female reproductive and endocrine diseases. (D) Organ-like engineering reconstruction is meaningful for the study of ovarian cancer transformation. (E) Microfluidics plays an important role in the exploration of ovarian *ex vivo* culture systems, with potential applications in fertility preservation and reproductive toxicology.

Additionally, cell-based methods have been employed to develop functional ovarian tissues from primordial germ cells (PGCs) and PGC-free gonadal cells in an ectopic xenogeneic environment. Hayama *et al.*' comparison of ovarian-like tissues generated from dispersed PGCs and PGC-free gonadal cells transplanted under the renal capsule of immunodeficient animals to normal gonads showed remarkable histological similarity. These induced xenograft models, capable of expressing oocyte markers Vasa and Stella, and yielding mouse antral follicle stage oocyte-like cells matured *in vitro* to metaphase II, highlight the potential of rat/mouse female PGCs and PGC-free gonadal cells to develop and reconstruct ovarian-like tissues containing functional oocytes in an ectopic xenogeneic microenvironment. This model holds promise as an invaluable tool for livestock breeding and human POF treatment research 91 (Figure 8A).

### 5.2. Hydrogels

Hydrogel, a semi-solid colloidal material, provides several functions for MSCs, including creating a 3D microenvironment closer to natural conditions, protecting cells from external stresses, and controlling the release of MSCs. Typically composed of a polymer network with a high water content, hydrogels possess characteristics such as softness, lightness, high water absorption, and moisture retention 92. In ovarian tissue engineering, hydrogel applications focus on delivery and encapsulation, such as using hydrogel encapsulation for tissue transplantation and serving as a "Trojan horse" for modulating drug release and enhancing targeted cell delivery. Hydrogel biomaterials pripremature ovarian failuremarily come in two types: natural materials like alginate, collagen, ECM, and hyaluronic acid, and synthetic materials such as synthetic polyesters including PLA, PGA, PCL, and PEG 93. Various types of hydrogels are applied in POF through four main approaches.

The first method employs hydrogel encapsulation for ovarian tissue transplantation. HRT is the most commonly used treatment for POF 94. HRT typically begins with a low dose of estrogen, followed by a combination of estrogen and progesterone therapy maintained until menopause. However, due to the lack of other ovarian hormones and lack of response to feedback regulation, this method leads to premature closure of the growth plates, cessation of bone growth, and long-term metabolic imbalance in women with POF 95. Moreover, gonadotoxic treatment and autologous cryopreserved ovarian tissue transplantation represent a promising new experimental method to restore fertility and ovarian endocrine function. However, due to the high sensitivity of the ovaries to radiotherapy and chemotherapy, a significant portion of POF patients originates from post-antitumor treatments, thus autologous tissue transplantation carries a risk of cancer recurrence 96. To mitigate the limitations of HRT and avoid the risk of cancer recurrence associated with autologous ovarian tissue transplantation from patients with POF due to radiotherapy and chemotherapy, many opt for hydrogel encapsulation and transplantation of ovarian tissue, as hydrogel-encapsulated ovarian tissue transplantation does not induce follicular apoptosis or immune rejection. Day *et al.* first demonstrated that ovarian tissue encapsulated in polyethylene glycol (PEG) hydrogel could prevent allogeneic transplant immune rejection. They encapsulated ovarian tissue from mice in PEG hydrogels with a degradable core and non-degradable shell. Compared to controls, the encapsulated tissue prevented sensitization to all allogeneic grafts without lymphocyte infiltration, proving that PEG-based hydrogels could serve as an immunological barrier for allogeneic ovarian tissue to restore mouse sex hormonal balance 97. Similarly, Gao *et al.* found that ovarian tissue encapsulated in fibrin hydrogel containing basic fibroblast growth factor (bFGF) significantly reduced the number of apoptotic follicles and improved the quality of ectopically transplanted mouse ovarian tissue 98. Tanaka *et al.* discovered that encapsulating ovarian tissue in gelatin hydrogel with bFGF could continuously release basic FGF, significantly increasing the density of primordial and primary follicles in frozen-thawed ovarian tissue grafts. 99.

The second method involves utilizing hydrogels to mimic the microenvironment. Currently, the culture of primordial follicles is primarily conducted through cortical tissue culture, also known as *in situ* culture, or via the culture of isolated follicles within a material matrix. Despite achieving promising results, *in situ* culture is challenging to control the follicular environment, and follicle growth within tissue fragments is limited, restricted to secondary follicles in size 100. Consequently, there has been a shift in focus from individual encapsulation to microenvironment culture 101. Felder *et al.* employed freeze-drying techniques to create robust, large-pore alginate scaffolds, which were then infused with bone morphogenetic protein-4 (BMP-4) to develop a synthetic ECM mimic platform for reconstructing the ovarian microenvironment for the ex-vivo maturation of primordial follicles. Results indicated an increase in the expression of genes related to follicular development, and after xenotransplantation of follicle devices supplemented with additional growth factors, follicles reached antral size and secreted sex hormones, restoring ovarian function in mice 102.

The utilization of hydrogels for stem cell delivery represents a third innovative method. Previously mentioned, MSC treatment for POF significantly promotes angiogenesis within the ovaries, reduces ovarian cell apoptosis, inhibits fibrosis, and regulates anti-inflammatory and immune responses, all of which are crucial for restoring ovarian function. Traditional methods of administration, such as orthotopic ovarian injection, tail vein injection, and intraperitoneal injection, have shown that direct ovarian injection yields the best results without a significant risk of tumorigenesis. However, this method requires at least a month for the transplanted stem cells to localize within the ovaries, leading to extended localization times and low transplantation rates 103. Hydrogels can enrich MSCs with target organs and provide them with an appropriate growth environment, thus enhancing the success rate of transplantation 104. Su *et al.* found that injecting MSCs together with a soluble collagen scaffold creates synergistic effects, resulting in a greater survival and accumulation of MSCs within the ovaries and aiding in the long-term recovery of ovarian functions, including the estrous cycle, estrogen levels, and follicle development 105. Furthermore, studies have demonstrated that MSCs encapsulated in sodium alginate bioglass within chemotherapy-induced POF models can protect granulosa cell functions and ovarian angiogenesis 106. Similarly, the transplantation of human UC-MSCs embedded in matrix gel or mounted on collagen scaffolds achieves the same therapeutic effects 107, 108. Shin *et al.* further discovered that localized delivery of embryonic stem cell-derived mesenchymal progenitor cells within hyaluronic acid gel increases ovarian reserves, estrogen, and anti-Müllerian hormone levels, ultimately improving the quality of oocytes and embryos in a simulated POF mouse model 109. Therefore, employing hydrogels for MSC delivery represents a highly promising candidate for the treatment of POF.

A fourth method involves utilizing hydrogels for controlled drug release. Drugs encapsulated in hydrogels are released in a controlled manner over an extended period, avoiding potential pharmacological hazards and inefficiencies associated with burst release. Hydrogels can also concentrate drugs within localized tissues, maintaining high drug concentrations and reducing adverse reactions. For example, directly inhibiting the mTOR activity offers a therapeutic approach for POF treatment but may also increase risks of diabetes and immune system impairment 110. Shi *et al.* developed an RTK-responsive hydrogel that judiciously releases an inhibitor to delay ovarian aging. The combination effectively enhances oocyte maturation and early embryonic development by downregulating mTOR activity, stimulating ovarian secretion of estrogen and progesterone, and developing more antral follicles, effectively delaying ovarian aging in aged mice. This innovative approach holds promise for enhancing TRAIL protein's therapeutic efficacy while minimizing adverse effects on normal cells. Overall, genetic engineering of MSCs transforms them into potent therapeutic vectors, enhancing treatment efficacy 111 (Figure 8B).

### 5.3. Bioprinting

The advantage of 3D printing lies in the ability to scale tissues to the size required by the recipient. Moreover, constructs can be printed with embedded vascular systems to mitigate the nutritional demands within large tissues 112. Research focuses within the realm of 3D bioprinting vary among different groups. The Ovsianikov group concentrates on innovating existing technologies, utilizing photosensitive organic-inorganic hybrid polymers ORMOCERs (ORganically MOdified CERamics) and epoxy-based SU8 materials, applying two-photon polymerization (2PP) technology to fabricate scaffolds with controllable topology and functionalities 113. Findings reveal that ORMOCERs did not improve doubling times or damage DNA but facilitated gap junction formation, and dual-photon polymerization of Ormocomp could adhere to vertical/steep surfaces to form layers after 3-4 days. These studies underscore the significant potential of 2PP technology in crafting scaffolds with precise topological structures and functionalities. Laronda's research aims to enhance different porosity angles to examine how changes in pore geometry achieved by manipulating the print layer's advance angle affect the survival of ovarian follicles. The findings suggest that scaffolds at 30° and 60° offer multiple surrounding angles for follicles, whereas 90° scaffolds have open porosity, limiting follicle-scaffold interactions. With increased scaffold interactions, follicle diffusion is restricted, survival rates rise, and ovarian function is fully restored upon implantation in mice. Furthermore, they developed a bioprosthetic ovary capable of releasing eggs without mechanical manipulation or digestible materials, highlighting the high functionality of such bioprosthetic ovaries using scalable and adaptable methods 114. Wu's team focuses on comparing the printability of different bioinks, finding that the 3D printing culture system using gelatin-methacryloyl exhibits favorable performance in hygroscopicity, degradation kinetics, and shape fidelity, presenting a viable alternative for follicle growth, development, and transfer, with broad clinical application prospects in female reproductive and endocrine diseases 115 (Figure 8C).

### 5.4. Organoids

Organoids are simple tissue engineering *ex vivo* models composed of assembled cells, which can be utilized to study tissue development, regeneration, and other fundamental human mechanisms. They also serve functions in disease diagnosis, modeling, personalized medicine, and functional research. 116. Although the specific causes of POF-related reproductive disorders remain unclear, maternal exposure to endocrine-disrupting chemicals in the environment is considered a contributing factor. Therefore, an appropriate *in vitro* gonadal development model system would enable a better understanding of these diseases and their origins. The three-layer gradient system (3-LGS) method allows for the generation of organized gonadal organoids within 7 days. Compared to models using complete tissue fragments, 3-LGS enables tracking of various cell populations and their interactions during development, examining the impact of exogenous factors on organogenesis, and easily manipulating cell groups by including or excluding them. This approach also supports organoid formation from pluripotent stem cells or primary cells from other target human tissues, offering additional model systems for regenerative medicine 117. Additionally, modeling TNF α-induced malignant phenotypes in normal human ovarian surface epithelial cell organoid models further supports the link between chronic inflammation and ovarian carcinogenesis 118 (Figure 8D). Organoid model plays a significant role in studying the pathogenesis of diseases.

### 5.5. Microfluidics

Microfluidics offers a new means of delivering DNA, RNA, proteins, and other biomolecules into cells. Traditional intracellular delivery methods, such as viral vectors and electroporation, fall short in maintaining cell viability, phenotype, functionality, and dosage. Microfluidics, with its superior controllability and scalability, achieves precise cellular manipulation at sub-cellular flow volumes, enabling efficient intracellular delivery 119. Nagashima utilized an innovative microfluidic dynamics system to support the *ex vivo* survival of feline and canine follicles enclosed within the ovarian cortex or isolated from it. Findings indicate that static conditions yield larger primordial follicles and support the transition of primordial to primary canine follicles but abnormally reduce RNA and GDF9 in cat follicles, suggesting species and tissue type differences in response to microfluidic culture. However, the culture system generally does not affect follicle development or oocyte health biomarker expression, representing an important exploration of improved *ex vivo* ovarian culture systems for large mammalian species, such as cats and dogs, with potential applications in fertility preservation, reproductive toxicology, and the conservation of endangered mammals 120 (Figure 8E).

## 6. Current clinical development status of MSC therapy in POF

### 6.1. Preclinical trials

In preclinical trials, animal models are commonly used to study the therapeutic effects of MSCs on POF. Several types of models are typically employed, including those induced by chemotherapeutic agents, autoimmune reactions, psychological stress, and substances like galactose, among others. Chemotherapy-induced models are classic tools for studying POF, with agents such as cyclophosphamide (CTX), busulfan (TG), cisplatin (CIS), and doxorubicin (DOX) being frequently used. These drugs mimic the pathophysiology of human POF by damaging cells in ovarian tissues, leading to diminished ovarian function. This method is relatively straightforward, allows for rapid model establishment, and visibly demonstrates ovarian damage. However, it comes with side effects, such as bone marrow suppression and hemorrhage (in CTX models), prolonged model formation time (in TG models), excessive toxicity leading to animal death (in CIS models), and inconsistent success rates (in DOX models). The autoimmune-induced model is the most relevant to the etiology of human POF. It primarily involves inducing an autoimmune response through thymectomy or other methods in neonatal mice to damage ovarian tissue. This model closely mirrors the pathogenesis of human POF, making it useful for studying disease mechanisms in depth. However, it presents challenges due to the complexity of the procedure, the difficulty of the surgery, and the high mortality rate. Additionally, the stability of the model remains uncertain and requires further validation. The psychologically induced model aligns with the mechanisms of POF development. This model induces ovarian dysfunction in animals through prolonged exposure to psychological stressors such as noise or restraint. While it effectively incorporates the role of psychological factors in POF pathogenesis, its drawbacks are significant: the model requires a long time to establish, and its stability has yet to be confirmed. The galactose animal model, meanwhile, offers a better simulation of the physiological aging seen in clinical POF patients. In this model, animals are administered galactose to induce ovarian dysfunction, thereby mimicking the aging characteristics observed in clinical POF cases. However, its success rate is relatively low, and the process is time-consuming 121. Lastly, the natural aging model closely replicates the decline in ovarian function during human aging and better simulates the physiological aging characteristics of clinical POF patients. It requires no artificial intervention to establish, thus avoiding potential side effects and trauma associated with other modeling methods. However, its limitations include a low success rate, as not all naturally aging mice exhibit typical POF symptoms. Additionally, the extended time required for mice to age naturally prolongs the research timeline and increases costs 122.

Additionally, the method of drug administration can impact model success. Common routes for inducing POF in mice include oral gavage, intraperitoneal, intravenous (tail vein), subcutaneous, and intradermal injections. Tail vein injection is the most commonly used method, allowing for unrestricted absorption, rapid onset, and buffering capacity, though it requires isotonic solutions, sterile conditions, and precise techniques. Intraperitoneal injection acts quickly but may cause discomfort. Subcutaneous and intradermal injections are convenient but vary in absorption rates, while oral gavage may be influenced by digestion. Each method has its pros and cons, with the choice depending on specific research needs and drug characteristics 123.

Following the evaluation of various modeling techniques, accurately assessing model success is crucial. Clear evaluation criteria ensure the reliability and validity of experimental results. Indicators of model success include reduced litter size, lower fertility index, changes in offspring number and average weight, and drastic weight loss in drug-induced models. Histological assessments reveal reductions in ovarian volume and weight, fewer corpora lutea and ovulations, extended estrous cycles, and changes in follicle numbers. Endocrinological markers show decreases in AMH and E2, alongside increases in FSH and LH. Granulosa cell biomarkers, including Ki67, Bcl2/Bax, Caspase 3/9, and FSHR, can evaluate ovarian proliferation and apoptosis levels 121. In summary, short-term indicators include reductions in antral/atretic follicles and corpora lutea, hormonal imbalances, and increased apoptotic markers, while long-term indicators include decreased fertility index and offspring number. These indicators help evaluate the success of MSC therapy in POF models and assess drug efficacy.

In conclusion, MSCs have demonstrated significant potential in the treatment of POF. Various modeling techniques offer crucial tools for understanding the pathological mechanisms of POF, while multiple delivery methods provide options for stem cell transplantation—though no universally accepted method exists, requiring careful evaluation of advantages and disadvantages based on specific conditions. Accurate assessment criteria ensure reliable evaluation of model establishment and treatment efficacy. As research into MSCs in POF animal models continues to advance, their therapeutic potential is increasingly promising. Numerous clinical trials are currently underway to further validate the feasibility and safety of MSCs in real-world clinical applications. Next, we will explore clinical trials of MSC therapy for POF.

### 6.2. Clinical trial

Currently, while the number of clinical trials involving MSC therapy for POF remains limited, some encouraging results have been reported. In these trials, MSCs are primarily transplanted via intravenous injection or intra-ovarian injection. Clinical trial outcomes have shown that MSC therapy can improve ovarian function, increase follicle numbers, enhance estrogen levels, reduce follicle-stimulating hormone (FSH) levels, and in some cases, even restore menstrual cycles and fertility (Table 8). However, several challenges and issues have also emerged. First, many trials have small sample sizes and short follow-up periods, highlighting the need to increase sample sizes and extend follow-up durations to fully assess the long-term efficacy and safety of MSC therapy for POF. Additionally, there is no clear consensus on the optimal dosage, transplantation route, or timing for MSC therapy in POF, which requires further exploration and refinement. Moreover, patient screening and evaluation in clinical trials need to be strengthened to ensure both the efficacy and safety of the treatment.

Notably, in an early clinical trial (NCT02696889), BM-MSCs were injected into the right ovary of patients with primary or secondary ovarian insufficiency, yielding significant results. The results showed that MSC transplantation was well-tolerated, with no reported adverse events. Regarding efficacy, hormone levels were monitored from 1 to 12 months post-surgery, showing a downward trend in serum FSH levels and upward trends in serum AMH and E2 levels. At 18 months, the data revealed a 50% reduction in serum FSH levels and a 30% increase in AMH and E2 levels, indicating significant hormonal improvement. Additionally, two patients experienced a single menstrual event 7 months post-procedure. Several ongoing studies further explore MSC therapy for POF. A Phase I trial has been initiated to evaluate the efficacy of locally injected AD-MSCs for treating POI, although it has not yet begun recruiting volunteers (NCT06132542). Another Phase I trial involving a single dose of UC-MSCs (Cordstem-ST) is currently recruiting participants (NCT06578039). Additionally, a Phase I trial assessing the safety and efficacy of intravenous YB-1113 for treating POI has been launched but is not yet recruiting (NCT05494723).

In terms of early clinical trials focusing on the use of engineered MSCs to treat POF, only one registered trial involves tissue-engineered MSCs. That study evaluates the adverse event rate and recovery outcomes following the injection of human umbilical cord MSCs (HUC-MSCs) combined with an injectable collagen scaffold into both ovaries to treat POF. Although this study has been completed, the results have not yet been published (NCT02644447).

### 6.3. Safety and ethics of stem cell therapy

MSCs demonstrate significant potential in treating POF, but safety and ethical concerns cannot be overlooked. While numerous preclinical studies emphasize ovarian function restoration and enhanced fertility, insufficient attention has been paid to the long-term safety and efficacy of such treatments. For genetically engineered MSCs or their exosomes, potential safety issues such as tumorigenicity and immunogenicity require special attention 124.

In terms of safety, while some scientists have raised concerns about the tumorigenic risks of MSCs, many studies suggest that MSC therapy is safe. Pei *et al.* assessed the safety of *in situ* MSC transplantation in a POF mouse model, concluding that MSCs did not exhibit tumorigenic properties, and no acute toxicity or significant immune reactions were observed, with minimal non-specific distribution 125. Similarly, Wang *et al.* conducted long-term monitoring after MSC transplantation to evaluate tumorigenicity, and despite the lack of a positive control group, their results supported the safety of *in situ* ovarian MSC transplantation 126. However, risks such as immune rejection or complications like cerebral infarction have been reported. For example, Zhu *et al.* found that in an experiment where cyclophosphamide-induced ovarian damage was treated with MSC tail vein injections, one rat developed cerebral infarction, highlighting potential safety concerns with MSC therapy 127. Additionally, Gao *et al.* discovered that MSCs derived from human lung cancer could promote tumor growth and immune suppression by inhibiting the anti-tumor activity of natural killer cells and T cells 128. Although current studies indicate a relatively high safety profile for MSC therapy in POF, long-term follow-up and monitoring remain essential to ensure patient safety.

From an ethical perspective, the clinical application of MSC therapy for POF must carefully consider the long-term effects on patients and their offspring, including ovarian function, fertility, tumor formation, and genomic alterations. In particular, when treating POF, the risks associated with genetically engineering MSCs to introduce exogenous genes, especially when oocytes are involved, must be carefully weighed 124. The source, acquisition, and use of stem cells require stricter ethical oversight. For example, the use of embryonic stem cells involves the destruction of embryos, sparking ethical controversy. Adult stem cells, such as MSCs, must be obtained and used in accordance with ethical guidelines, ensuring informed consent and patient rights protection to avoid a recurrence of incidents like the “gene-edited babies” scandal.

China has introduced several policies to promote stem cell research, regulated mainly by the *Measures for Ethical Review of Biomedical Research Involving Humans* and the *Ethical Guidelines for Research on Human Embryonic Stem Cells*. However, the *Ethical Guidelines for Research on Human Embryonic Stem Cells* are brief and lack specific guidance and punitive measures, leaving the regulatory framework relatively lax and in need of further improvement 129. Additionally, the commercialization and market development of stem cell therapies require more stringent policies to prevent unregulated treatment practices and misleading advertising, while also avoiding over-regulation that could stifle industry growth.

In summary, the exploration of MSC therapy for POF must carefully address safety and ethical issues, establish comprehensive regulatory frameworks, and strengthen ethical review systems to ensure the sustainable development and widespread adoption of this promising treatment.

## 7. Conclusion and perspective

In the realm of regenerative medicine, the further exploration of MSC functional subpopulations and engineering modifications presents a paradigm shift for the standardized treatment of POF. Characterized by diminished ovarian function before the age of 40, POF poses a significant challenge to reproductive medicine due to the limited treatment options available. The pluripotent nature of MSCs, encompassing their differentiation, immunomodulatory, and regenerative capabilities, positions them as viable candidates for therapeutic intervention. However, the clinical application of MSCs is hampered by their heterogeneity, complicating the establishment of standardized treatment protocols.

The application of scRNA-seq offers a new avenue for dissecting this heterogeneity, enabling the identification of distinct MSC subpopulations with specific gene expression profiles and functional characteristics. This detailed analysis aids in selecting MSC subpopulations with optimal performance for POF treatment, enhancing their homing, proliferation, and differentiation capabilities within ovarian tissue. It's noteworthy that the research focus of different groups on MSC subpopulations has led to nomenclature heterogeneity rather than the existence of multiple distinct subpopulations. To date, only a handful of studies have successfully isolated subpopulations with specific functions, mainly focusing on wound healing capabilities.

Furthermore, the advancements in genetic and tissue engineering also offer exciting possibilities for enhancing the efficacy of MSC therapies. This paper, through existing research, elaborates on the genetic engineering modifications of MSCs surrounding the improvement of their innate functions, such as enhancing MSC proliferation and differentiation, improving MSC migration and homing, enhancing MSC adhesion, delaying premature aging of MSCs, increasing MSC survival rates, and bestowing new functions like acting as carriers and therapeutic switches. It also details effective strategies for tissue engineering modifications of MSCs to enhance the regenerative potential of ovarian tissue from different materials perspectives. The application of engineering modification techniques is expected to improve the existing capabilities of MSCs and overcome the limitations brought by the inherent characteristics of MSCs, paving the way for the advancement of standardized clinical treatment protocols for MSCs.

Moreover, the integration of preclinical and clinical trial data, alongside an examination of the safety and ethical considerations of MSC therapy for POF, is critical. The paper addresses these aspects to ensure that MSC-based treatments not only advance scientifically but also adhere to rigorous safety standards and ethical guidelines. The goal is to transition from laboratory successes to clinically effective and ethically sound therapies.

In summary, the promising outcomes of MSC therapy for POF underscore the potential of stem cell-based therapies in addressing unmet clinical needs. However, bridging the gap from laboratory to bedside for MSC therapy in POF remains a lengthy journey. With advancements in this field, including ongoing preclinical and clinical trials, and a focus on safety and ethical considerations, it is hoped that MSC therapy can provide a viable and effective treatment option for patients with POF.

## Figures and Tables

**Table 1:** Boosting proliferation and differentiation of MSCs

	Mode of action	Effects on	Cell Source	Method	References
HGF	Increased IGF-1 secretion and miR-101-3p, ETV1 expression	Suppressing osteogenic differentiation	hMSCs	Treating MSCs with IGF-1	130
Sox9	Promoted BMP2 expression by downregulating Smad7 signaling pathway	Augmenting Chondral differentiation	MSCs	Adenoviral vector transfection	131
NOS2	Upregulated the JAK/STAT3 signaling pathway	Augmenting lipogenic differentiation	Rat MSCs	Treating MSCs with DMEM	132
GDF11	Upregulated the TGF-β -R/ERK/EIF4E signaling pathway	Augmenting endothelial differentiation	Mouse BM-MSCs	Lentiviral vector transduction	133
FOXO1	Upregulated TGF-β1/SMAD signaling pathway and increased COL2A1, ACAN, SOX9, P21 expression	Suppressing chondral differentiation	hMSCs	Treating MSCs with FBS to create 3D pellets	134
microRNA-23c	Downregulated FGF2, COL2, ACAN expression	Suppressing chondral differentiation	Rat BM-MSCs	Co-transfection of microRNA-23c mimic and FGF2 overexpression plasmid	135
IL-3	Upregulated JAK/STAT signaling pathway, promoted BMP-2, Smad1/5/8, osterix and RUNX2 expression	Augmenting osteogenic differentiation	hBM-MSCs	MSCs were cultured in osteogenic medium containing IL-3	136
miR-223-3P	Promoted FOXO3, RUNX2, OCN, Smad4 expression	Augmenting osteogenic differentiation	hBM-MSCs	Lentiviral vector transfection	137
Oct-4	Upregulated CIP2A signaling pathway	Augmenting chondral differentiation	hMSCs	Lentiviral vector transfection	138
Oct4/Sox2	Promoted Cyclin D1 expression	Augmenting Lipogenic differentiation and osteogenic differentiation, proliferation	hAD-MSCs	Treating MSCs with Oct4/Sox2-containing plasmid and D-fection complex	139
SOD2	Downregulated mtROS expression	Suppressing lipogenic differentiation	hAD-MSCs	Treating MSCs with IFN-γ and TNF-α	140
p53	Declined osterix expression	Suppressing osteogenic differentiation and proliferation	Mouse BM-MSCs	Getting MSCs from P53 gene knockout (KO) mice	141
TGF-β1/NGF	Upregulated PI3K-AKT signaling pathway	Augmenting chondral differentiation	BM-MSCs	Treating MSCs with TGF-β1	37
LECT2	Downregulated the Wnt/β-catenin signaling pathway	Suppressing Osteogenic differentiation	hBM-MSCs	Transfecting LECT2 siRNA into MSCs	142
EGF	Delayed activation of β-catenin signaling pathway	Regulating proliferative	hMSCs	Lentiviral vector transduction	143
DKK1	Upregulated Wnt/β-catenin signaling	Augmenting Osteogenic differentiation, proliferation	BM-MSCs	Lentiviral vector transfection	144
TIMP-1	Downregulated Wnt/β-catenin signaling	Suppressing proliferation and osteogenic differentiation	hBM-MSCs	Lentiviral vector transfection	145
miR-10b	Downregulated TGF-β/SMAD2 signaling pathway	Augmenting osteogenic differentiation and suppressing adipogenic differentiation	hAD-MSCs	Lentiviral vector transfection	146
LncRNA-CIR6	Promoted CDK1	Augmenting myocardial differentiation	hUC-MSCs	Transfecting LncRNA-CIR6 into MSCs	147

**Abbreviations:** GDF11: growth differentiation factor 11; CDK1: cyclin-dependent kinase 1; AD-MSC: adipose tissue-derived MSCs.

**Table 2:** Enhancing migration and homing abilities of MSCs

	Mode of action	Effects on	Cell Source	Method	References
SDF-1	Upregulated SDF-1/CXCR4 signaling pathway	Increasing migration and homing to the bone defect area	Rat MSCs	Lentiviral vector transduction	148
TGF-β1	Upregulated SDF-1/CXCR4 signaling pathway	Enhancing homing at sites of myocardial injury	Rat MSCs	Culturing MSCs with anti-TGF-β1	149
DFO	Upregulated PI3K/AKT and SDF-1/CXCR4 signaling pathway	Increasing migration and homing of MSCs to the injured cochlea	Rat BM-MSCs	Culturing MSCs with DFO	150
CM	Prometed AQP1, CXCR4 expression and upregulated FAK, Akt and Erk signaling pathway	Enhancing migration of oMSCs	Ovine BM-MSCs	Culturing MSCs with FBS	45
FOXQ1	Declined p16,p21,p53 expression, promoted SIRT1, PCNA expression	Increasing hUC-MSC migration *in vivo* and *in vitro*	hUC-MSCs	Lentiviral vector transduction	151
AT2R	Declined IL-1β, IL-6 expression, promoted IL-10 expression	Increasing migration	hBM-MSCs	Lentiviral vector transduction	152
CXCR4/IL-35	Declined IFN-γ, IL-4 and IL-17A expression ,promoted IL-10 expression	Increasing migration of MSC	Rat BM-MSCs	Lentiviral vector transfection	153
CXCR7	Downregulated Wnt/β-catenin signaling pathway to declined Notch/Jag1 expression	Increased homing efficiency of MSC	hUC-MSC	Lentiviral vector transduction	154
CCR2	Declined TNF-α, IL-6, and IL-1β expression	Enhanced migration of MSCs to damaged liver	hUC-MSC	Lentiviral vector transduction	155
CSF2RB	Upregulated STAT5/RNF4 signaling pathway	Promoting MSC migration to the heart	Mouse AD-MSC	Adenoviral vector transfection	156

**Abbreviations:** DFO: deferoxamine; oMSCs: ovine mesenchymal stem cells; PCNA: proliferating cell nuclear antigen; AT2R: angiotensin II type 2 receptor; SDF-1: stromal cell-derived factor 1; CSF2RB: colony-stimulating factor 2 receptor beta subunit.

**Table 3:** Enhancing adhesiveness of MSCs

	Mode of action	Effects on	Cell Source	Method	References
PMA	Promoted PKC to upregulated FAK and Rho/Rac-1 signaling pathways	Enhancing cell adhesion	MSCs	Treatment of MSCs with PKC	157
PAI-1	Upregulated vitronectin expression	Directly improving MSC adhesion	Mouse MSC/BM-MSCs	Integrating retroviral vector transduction	158
ILK	Upregulated PKB/Akt and ERK signaling pathways	Promoting cell survival and adhesion and ameliorated myocardial injury	Rat BM-MSCs	Lentiviral vector transduction	159
miR-9-5-p	Upregulated β-catenin signaling pathway	The formation and distribution of focal adhensions as well as the reorganization of F-actin	Rat BM-MSCs	Treatment of MSCs with HGF	160
SDF-1	Upregulated CXCR4 to promote Akt signaling pathway; upregulated CXCR4 to promote eNOS and VEGF expression	Promoting myocardial angiogenesis and prevent myocardial infarction	Rat BM-MSCs	Adenoviral vector transfection	161
tTG	Promoted FAK, Src, PI3K phosphorylation	Increasing MSC adhesion as well as cell viability	Rat BM-MSCs	Transfection of eukaryotic expression pMT2 vector	162
IL-1β	Promoted integrin α5β1 expression	Enhancing MSC adhesion	hBM-MSCs	Incubation of MSCs with IL-1β in the medium	47
Tropoelastin(TE)	Upregulated integrin-β1/ERK/VCL signaling pathway	Enhancing survival and adhesion of MSCs	hIPFP-MSCs	Suspension of MSCs in TE solution	163
ITGA10	Upregulated FAK/PI3K/AKT/GSK3β/β-catenin signaling pathway	Enhanced adhesion and osteogenic differentiation of MSCs	BM-MSCs	Lentiviral vector transduction	49

**Abbreviations**: FAK: focal adhesion kinase; PKC: protein kinase C; eNOS: endothelial nitrous oxide synthase; PHD2: prolyl hydroxylase domain-containing 2.

**Table 4:** Decelerating premature senescence in MSCs

	Mode of action	Effects on	Cell Source	Method	References
Rapamycin (mTOR specific inhibitor)	Inhibited mTOR signaling, suppressed p16^INK4A^ protein expression, reduced secretion of IL6, increased expression of NANOG	Postponing replicative senescence of BM-MSCs	hBM-MSCs	Culturing MSCs in rapamycin's medium	164
Melatonin	Attenuated mTOR signaling pathway, activated Akt signaling pathway	Ameliorating PC-induced senescence of MSCs	hAD-MSCs	Pre-incubation of MCS in PC containing melatonin	58
IGF-1	Upregulated Akt/mTOR signaling pathway	Influencing apoptosis and autophagy in aged BM-MSCs, affecting their tolerance to hypoxia and survival after transplantation in myocardial infarction	Mouse BM-MSCs	Transfection of MSC with IGF-1-specific siRNA	165
Licochalcone D (Lico D)	Upregulated AMPK signaling pathway	Improving oxidative stress-induced senescence of MSC	hBM-MSCs	Treating MSC with Lico D	166
Apelin	Activated AMPK signaling pathway	Rejuvenating aged MSCs, enhancing their paracrine effects and improving cardiac protection after infarction	hBM-MSCs	Lentiviral vector transfection	167
PBX1	Downregulated ROS expression and attenuated ROS-mediated DNA damage	Attenuating ROS-mediated DNA damage and delaying senescence and apoptosis of HF-MSCs	HF-MSCs	Lentiviral vector transfection	54
Sirt3	Downregulated ROS and upregulated SOD2 expression and activity	Reducing oxidative stress-induced rat BM-MSC senescence	Rat BM-MSCs	Lentiviral vector transfection	168
STC1	Downregulated ROS expression	Attenuating ROS-mediated effects and delaying senescence of hTMSCs	hTMSCs	Transfection by siRNA	169
IHH	Downregulated ROS/mTOR signaling pathway	Slowing the aging of BM-MSCs	BM-MSCs	Transfection with IHH siRNA	170
Nampt	Upregulated Sirt1 expression and intracellular NAD concentrations	Attenuates cellular senescence in senescent MSCs	Rat BM-MSCs	Lentiviral vector transfection	171
FOXQ1	Upregulated Sirt1and PCNA expression, downregulated p16, p21, p53 expression	Enhancement of MSC resistance to ageing and migration	hUC-MSCs	Lentiviral vector transfection	151
ALKBH5	Increased m6A modifications, reduced CDKN1C expression	Rejuvenation of senescent MSC when downregulated	hBM-MSCs	Lentiviral vector transfection	172
p300	Downregulated p53/p21 signaling pathway	Inhibition of MSC senescence	hUC-MSCs	Transfection with p300-targeted siRNAs	61
miR-873-5p	Regulated AMPK signaling pathway and upregulated Cab39 expression	Rejuvenation of senescent MSCs when inhibited	hMSCs	Transfection	173
ERRα (the potential target of genistein)	Upregulated sirt3 and PGC1α expression	Attenuating premature senescence in rat BM-MSCs	Rat BM-MSCs	Transfection with ERRα-targeted siRNAs	174
miR-1292	Activated Wnt/β-catenin signaling pathway and upregulated FZD4 expression	Slowing MSC senescence and promotes MSC osteogenic differentiation when inhibited	hAD-MSCs	Transfection with miR-1292 siRNAs	175
miR-146a	Downregulated TRAF6/NF-κB signaling pathway	Reducing MSCs senescence when upregulated	hBM-MSCs	Lentiviral vector transfection	176
miR-155-5p	Upregulated Cab39/AMPK signaling pathway	Rejuvenating AMSCs when downregulated	hBM-MSCs	Lentiviral vector transfection	177
miR-34a	Upregulated Nampt expression and mediated by the NAD^+^-Sirt1 pathway	Reversing senescence when suppressed	Rat BM-MSCs	Lentiviral vector transfection	56
PBX1	Upregulated SIRT1 expression, downregulated PARP1 expression	Alleviating HF-MSCs senescence and apoptosis	HF-MSCs	Transfection by siRNA	178
miR-483-3p	Upregulated IGF1 expression	Retarding the adipogenic differentiation potential of hAD-MSCs and reducing cellular senescence when knocked down	hAD-MSCs	Cell transfection	60

**Abbreviations:** GLEXG: GuiLu-ErXian glue; Lico D: licorice chalcone D; Nampt: nicotinamide phosphoribosyltransferase; hTMSCs: human palatine tonsil MSC.

**Table 5:** Improving MSC survival rates

	Mode of action	Effects on	Cell Source	Method	References
miRNA-34a	Upregulated Bcl-2 and Ki67 mRNA expression	Increasing MSC survival in hypoxic environments when inhibited	hBM-MSCs	Transfection with anti-34a	62
RIPK1	Suppressed the activation of the p53-PUMA signaling pathway	Improving the survival of MSCs	Rat BM-MSCs	Transfection with siRIPK1	179
HIF1A	Downregulated p53 signaling pathway	Improving MSC survival	Rat BM-MSCs	pcDNA3.1 vector for transfection	74
VEGF/Bcl-2	Promoted p62 expression, Suppressed LC3II/I, Beclin-1 and cleaved-caspase-3 expression	Improving MSC survival when co-overexpressed VEGF and Bcl-2	Rat MSCs	Lentiviral vector transfection	68
ILK	Upregulated the phosphorylation of AKT and mTOR	Improvement of MSC survival	Rat BM-MSCs	Transfection with ILK-siRNAs	64
PKCɛ	Enhanced SDF-1/CXCR4 signaling pathway and PI3K/AKT signaling pathway activity	Improving MSC survival	Rat BM-MSCs	Lentiviral vector transfection	180
Gas6	Upregulated PI3K/Akt signaling pathway	Improvement of MSC survival rate	Rat BM-MSCs	Adenoviral vector transfection	72
Gremlin1	Upregulated the PI3K/Akt signaling pathway	improvement of MSC survival rate	hBM-MSCs	Lentiviral vector transfection	70
SDF-1/CXCR4 axis	Promoted Akt and Erk signaling pathway, upregulated Bcl-2/Bax ratio	Improvement of MSC survival and proliferation rate	Rat BM-MSCs	SDF-1 pretreatment MSC	181
ILK	Promoted lncTCF7/Wnt pathway	Enhancing MSC survival and self-renewal	Rat BM-MSCs	Recombinant adenoviral vector transfection	65
HGF	HGF-eMSCs secreted HGF to prime BM-MSCs, upregulated paracrine of BM-MSCs	Prolonging survival of BM-MSC	hBM-MSC	Lentiviral vector transfection of MSC	182
TNFR2	Upregulated TNFα/TNFR2 signaling pathway	Improving MSC survival	Mouse BM-MSCs	TNFR2 knockout (TNFR2 KO) mice	71
IL-13	Switched RMG from M1 to M2, reduced MHC II and pro-inflammatory cytokines	Improvement of MSC survival rate	Rat BM-MSCs	Lentiviral vector transfection	73
Forskolin (Fsk)	Upregulated TrkB expression and BDNF worked synergistically with upregulated TrkB by Fsk	Improving survival of hBM-MSCs	hBM-MSC	Treatment of hBM-MSCs with Fsk	75

**Abbreviations:** BDNF: brain-derived neurotrophic factor; RMG: retinal microglia.

**Table 6:** Acting as a therapeutic switch

Agent	Mode of action	Effects on	Cell Source	Method	References
Steroids (budesonide)	Upregulated FOXO3 to promote indoleamine-2,3-dioxygenase (IDO)	Increasing MSC immunomodulation	hMSCs	Particle modification	183
curcumin	Downregulated PTEN/P53/Caspase-3 signaling pathway and upregulated AKT and HO-1 signaling protein expression	Enhancing myocardial repair in MSCs	Rat AD-MSCs	Curcumin Pretreated MSCs	184
NurOwn	Induced secretion of high levels of NTFs; upregulated IFN-γ, IL-6, TNF-α and so on	Treatment of amyotrophic lateral sclerosis (ALS)	BM-MSCs	NTF treated MSCs	80
VPA+S1P	Activation of MAPK^p42/44^ signaling pathway; AKT signaling pathway; and upregulated SDF-1/CXCR4 signaling pathway	Promoting migration, proliferation, self-renewal and anti-inflammatory capacity of MSCs	hUC-MSCs	VPA+S1P or 5-Aza Treated MSCs	79
LL-37	Upregulated TLR3 levels	Promoting the migration of hPD-MSCs	hPD-MSCs	LL-37 incubated MSCs	185
DSP30	Upregulated TLR4, IL-1β, IL-6; TLR9, TGF-β1 expression and downregulated TNF-α	Enhancing the immunosuppressive properties of MSCs	BM-MSCs	DSP30 treated MSCs	186
TPCA-1	Downregulated pro-inflammatory factors such as IL-6 and MCP-1	Inhibition of myocardial fibrosis	hMSCs	TNF-α and TPCA-1 treated MSCs	187
IFN-γ	Upregulated IFN-γ-Janus kinase (JAK) and activator of transcription 1 (STAT1) signalling pathways	Reduced symptoms of graft-versus-host disease (GVHD) in NOD-SCID mice	hMSCs	Lentiviral vector transfection	188
TNF-α	Upregulated the NF-κB/COX2 signalling pathway to promote PGE2 expression	Enhancing immunomodulation and induction of osteogenic differentiation	hMSCs	LPS plus TNF-α pretreated MSCs	189

**Abbreviations:** IDO: indoleamine-2,3-dioxygenase; ALS: amyotrophic lateral sclerosis; pMSCs: placenta-derived MSCs; GVHD: graft-versus-host disease.

**Table 7:** As a vehicle to improve therapeutic efficacy

Disease type	Source	Method	Delivery	Effect	References
PF	AD-MSCs	Specific biological coupling	Nintedanib	Antifibrotic effect	190
CMV pneumonia	BM-MSCs	Membrane coating	GCV or PFA	Suppressing inflammation	191
Prostate cancer	BM-MSCs	Particle labeling	MNPs	Anti-tumor proliferation	192
Colon Tumor	AD-MSCs	Metabolic glycoengineering and copper-free click chemistry	AuNPs	Enhancing photothermal effect	193
Tumor	C3H10T1/2	Avidin-biotin complex method	DOX-Lips	Enhancing the intercellular delivery of DOX	194
Parkinson's disease	BM-MSCs	Viral transduction	Overexpression of GDNF	Providing localized neuroprotection in an inflammation-driven rat model of Parkinson's disease	82
HD	hMSCs	Lentiviral transduction	Overexpression of BDNF	Improving Outcomes in Huntington's Disease Mouse Models by reducing striatal atrophy in YAC128 mice	83
VCF	BM-MSCs	Plasmid transfection	Overexpression of BMP6	Inducing bone regeneration	195
EAE	AD-MSCs	Lentiviral transduction	IFN-β	Ameliorating the symptoms of MS in EAE models and reducing indications for peripheral and central neuroinflammation	196
Stem cell immunosuppression	hMSCs	Particle modification	Budesonide	Enhances the inhibitory effect of stimulated peripheral blood mononuclear cells	183
Liver fibrosis	BM-MSCs	Adenovirus transfection	DCN	Inhibits the rat liver fibrosis induced by thioacetamide	197
Myocardial infarction	MSCs	Glandular carrier modification	Trx1	Increased pro-angiogenic factors, reduced fibrosis and improved heart function in the infarcted rat myocardium	198
B-ALL	hUC-MSCs	Viral transduction	Expression of TRAIL	Inhibit the growth of B-ALL cells and ease the spleen and kidney injury induced by B-ALL	199

**Abbreviations:** PF: pulmonary fibrosis; CMV: cytomegalovirus; GCV: ganciclovir; PFA: phosphonoformate; MNPs: magnetic nanoparticles; AuNPs: gold nanoparticles; DOX-Lips: doxorubicin-loaded liposomes; HD: Huntington's disease; VCF: vertebral compression fractures; EAE: experimental autoimmune encephalomyelitis; B-ALL: B-cell acute lymphoblastic leukemia; TRAIL: TNF-associated apoptosis-inducing ligand; HO-1: heme oxygenase-1; Trx1: thioredoxin-1.

**Table 8:** Clinical trials of engineered modified or unmodified MSCs for the treatment of POF

Start date	Purpose	Enrollment	MSC types	MSCs surgical transplantation approach	Types of patients with POF	Outcome of trials	Phases	Study status	Trial ID
Mar-2012	MSC therapy combined with hormone replacement therapy for POF	40	hUC-MSCs	Ovarian injection	Patients with POF undergoing hormone replacement therapy	No results posted	Phase1/Phase2	Unknown	NCT01742533
Mar-2012	Autologous MSCs transplantation for the treatment of POF in women	60	BM-MSCs (suspended in PRP)	Ovarian laparoscopic injection	Patients with POF and FSH levels ≥ 20 IU/L	Reduced serum FSH levels, increased estrogen and AMH levels; disappearance of menopausal symptoms	Phase1/Phase2	Unknown	NCT02062931
Mar-2012	Effect of autologous MSC therapy for POF on pregnancy	112	BM-MSCs	Ovarian laparoscopic injection	Patients with POF	No results posted	Phase1/Phase2	Completed	NCT02372474
Jan-2014	Autologous MSC transplantation for idiopathic and drug induced POF	60	hBM-MSCs	Ovarian injection	Patients with idiopathic and drug induced POF	Reduced serum FSH levels, increased estrogen and AMH levels; disappearance of menopausal symptoms	Phase1/Phase2	Unknown	NCT02043743
Jan-2015	Autologous bone marrow-derived stem cell transplantation for POF	50	BM-MSCs	Ovarian injection	Patients with POF with FSH more than 20 IU/L	No results posted	Phase1/Phase2	Unknown	NCT03069209
Sep-2015	Autologous adipose-derived mesenchymal stem cell transplantation for POF	9	AD-MSCs	Ovarian injection	Patients 20 to 39 years of age with FSH more than 20 IU/L	No results posted	Phase1/Phase2	Unknown	NCT02603744
Oct-2015	hUC-MSCs with injectable collagen scaffold transplantation for POF	23	hUC-MSCs	Homozygous hUC-MSCs bilateral ovarian injection	Patients with POF show no response to drug therapy	No results posted	Phase1/Phase2	Completed	NCT02644447
Feb-2016	hUC-MSCs transplantation for POF	320	hUC-MSCs	Ultrasound-guided ovarian injection	Patients with POF	No results posted	Phase1/Phase2	Unknown	NCT03033277
Feb-2016	MSC therapy for POF	3	BM-MSCs	Ovarian Laparoscopic injection	Patients with primary or secondary POF	50% decrease in serum FSH values; 30% increase in serum AMH and E2 values; resumption of menses; improvement in estrogen levels toward normal ranges; achievement of pregnancy	NA	Completed	NCT02696889
Oct-2018	Investigating the safety and efficacy of MSC therapy for POF	12	hUC-MSCs	Intravenous injection	Patients with POF without hormone therapy and Chinese medicine within 3 months	No results posted	Phase2	Suspended	NCT03816852
Dec-2018	UCA-PSCs treatment of POF	20	WJ-MSCs	Ultrasound-guided ovarian injection	Patients with POF who have received a nonphysiologic hormone replacement program and have not response to pharmacologic therapy	No results posted	Phase1	Completed	NCT05138367
Apr-2019	MSCs transplantation treatment of POF	28	hESC-MSCs	Ultrasound-guided bilateral ovarian injection	Patients with early onset ovarian hypoplasia.	No results posted	Phase1	Unknown	NCT03877471
Nov-2019	Study of hUC-MSCs to treat POF	66	hUC-MSCs	TVUS-guided bilateral ovarian injection	Patients who meet the POF diagnostic criteria and have no spontaneous follicular activity	No results posted	NA	Unknown	NCT05308342
Jan-2020	Study of the application of amplified MSC to POF	10	BM-MSCs	Ovarian injection	Patients with POF aged 18 to 38 years	No results posted	Phase1	Unknown	NCT04815213
Jan-2024	AD-MSCs transplantation treatment of POF	10	AD-MSCs	Ultrasound-guided ovarian injection	Patients with POF	No results posted	Phase1	Not yet recruiting	NCT06132542
Aug-2024	Study CordSTEM-ST treatment of POF	6	CordSTEM-ST (homozygous UC-MSCs)	Single dose administration	Patients with POF who are older than or equal to 25 years and younger than 40 years of age with FSH more than or equal to 40 IU/L	No results posted	Phase1	Recruiting	NCT06578039
Jan-2025	Study of the safety and efficacy of YB-1113 in the treatment of POF	6	YB-1113 (hUC-MSCs)	Intravenous injection	Patients with POF with FSH more than or equal to 25 IU/L who are older than 18 years and younger than 40 years of age	No results posted	Phase1	Not yet recruiting	NCT05494723

**Abbreviations:** PRP: platelets rich plasma; AMH: anti-Mullerian hormone.
